# Phenylseleninate-Connected Telluroxane Clusters

**DOI:** 10.1021/acs.inorgchem.4c02274

**Published:** 2024-08-26

**Authors:** Jéssica F. Rodrigues, Ana Júlia Z. Londero, Bárbara Tirloni, Maximilian Roca Jungfer, Ulrich Abram, Ernesto S. Lang

**Affiliations:** † Departamento de Química, Universidade Federal de Santa Maria − UFSM, Laboratório de Materiais Inorgânicos − LMI, 97105-900, Santa Maria, RS Brazil; ‡ Ruprecht-Karls Universität Heidelberg, Im Neuenheimer Feld 271, D-69120 Heidelberg, Germany; § Institute of Chemistry and Biochemistry, Freie Universität Berlin, Fabeckstr. 34-36, D-14195 Berlin, Germany

## Abstract

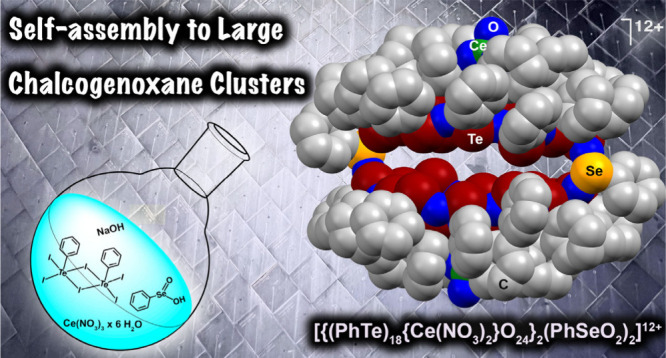

Hydrolysis of PhTeI_3_ in the presence of sodium phenylseleninate and M^3+^ ions (M = Y, Nd, Ce) gives well-defined, bowl-shaped telluroxane clusters. Each of the two half-spheres of the compositions [(PhTe)_18_{ML}O_24_]^7+/8+^ ({ML} = {Y(NO_3_)(H_2_O)}^2+^ (**1**), {Nd(NO_3_)(H_2_O)^2+^} (**2**), or {Ce(NO_3_)_2_}^+^ (**3**)) are connected by two (compound **3**) or four (compounds **1** and **2**) PhSeO_2_^–^ bridges. The resulting chalcogenoxane spheres have internal volumes of approximately 1500 Å^3^. Charge compensation is provided by nitrate ions and/or [Na_2_(NO_3_)_8_]^6–^ clusters, which are located inside and surrounding these spheres.

## Abstract 2

Large, spherical chalcogenoxane clusters are formed by hydrolysis of [PhTeI_3_]_2_ in the presence of sodium phenylseleninate and M^3+^ nitrates (M = Y, Nd, Ce). These clusters comprise two M^3+^-centered telluroxane bowls being connected by phenylseleninate bridges. The voids inside these spheres have volumes of approximately 1500 Å^3^ and are filled with nitrate and/or [Na_2_(NO_3_)_8_]^6^ counterions.

Organotelluroxanes are compounds with at least one covalent tellurium–carbon bond and more or less defined extended networks of Te–O bonds. They are usually formed by hydrolysis of respective organotellurium halides.^1−3^ Particularly, the synthesis of corresponding tellurium(IV) compounds requires strict control of the reaction conditions and/or the use of sterically encumbered or chelating organic substituents, which prevents rapid and uncontrolled polymerization and allows the isolation of defined molecular aggregates. Following such strategies, several examples of crystalline telluroxanes could be isolated during the past decade.^3−10^ The structures established by such compounds comprise well-defined linear polymers and cyclic systems, with a varying number of aryl-TeO units building their backbones. Some examples are shown in Chart 1. A remarkable structural motif is found in compounds containing two [(PhTe)_19_O_24_]^9+^ half-shells, which are connected by a layer of 18 bromide or iodide ions (Chart 1d).^11−13^ Such compounds were first found as unintended products of disproportionation of the selenone adduct [PhTe(1,3-dibutylbenzimidazoline-2-selenone)][PhTeBr_2_] in acetonitrile and subsequent hydrolysis.^12^ Alternatively, they were formed from reactions of 3-(PhTe)propylamine or {3-(PhTe)propyl}picolinamide with iodine and subsequent hydrolysis.^11,13^ More controlled access to such products succeeded by use of [PhTeI]_4_ or [PhTeI_3_]_2_ as starting materials.^13^

**Chart 1:**
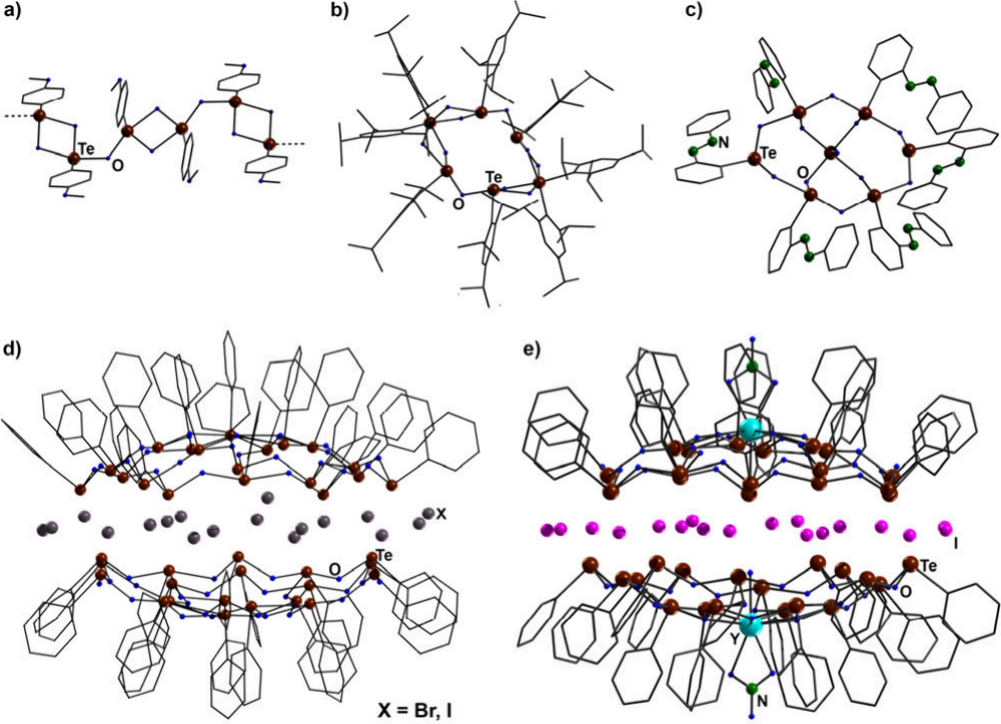
Examples of Well-Defined Telluroxanes: (a) [(4-MeOPhTeO)_2_O]_∞_,^5^ (b) [(2,4,6-Me_3_PhTeO)_2_O]_6_,^6^ (c) [(1-PhNN)PhTeO)_6_(TeO_5_)],^8^ (d) [{(PhTe)_19_O_24_}_2_X_18_] (X = Br, I),^11,12^ and (e) [{(PhTe)_18_O_24_Y(NO_3_)(H_2_O)}_2_I_16_]^13^

A modification of the basic structure of the [(PhTe)_19_O_24_]^9+^ cluster is possible by replacement of the central {PhTe}^3+^ unit by Ca^2+^ or M^3+^ ions (M^3+^ = Y^3+^ or Ln^3+^; Chart 1e). This results in the formation of “functionalized” telluroxanes with modified optical and magnetic properties.^13^ The role of the central halide layer in the formation and the stabilization of the spherical units is not yet completely clear. From our previous studies, it can be concluded that at least the iodide compounds [{(PhTe)_19_O_24_}_2_I_18_], [{(PhTe)_18_O_24_M(NO_3_)(H_2_O)}_2_I_16_] (M = Y, La, Eu, Lu), and [{(PhTe)_18_O_24_Ca(H_2_O)_2_)}_2_I_16_] are stable entities. They are transferred to the gas phase without decomposition, as is evidenced by their mass spectra,^13^ whereas an inherent instability is reported for the corresponding bromide cluster.^12^

In continuation of this work, we are interested in the synthesis of more “metal-decorated” compounds and in the properties of the products. Of particular interest is also the role of the established halide layers in the stabilization of such aggregates.

In previous studies, we could show that the positions of the halide ions within the layer between the telluroxane clusters are flexible and the voids between the half-spheres can accommodate additional solvent molecules such as methanol or pyridine.^11,13^ Furthermore, the number of iodide ions in such layers is variable. They provide charge compensation and, thus, depend on the positive charge of the telluroxane skeletons.^11,13^

Here, we describe syntheses, spectroscopic characterization, and structures of spherical telluroxane cluster cations without central halide layers. Such compounds are formed in self-assembled reactions from mixtures of [PhTeI_3_]_2_, phenylseleninic acid, M(NO_3_)_3_·6H_2_O (M = Y, Nd, Ce) salts and NaOH (Scheme 1) in dioxane. The products precipitated as golden-yellow solids from the reaction mixtures. Unlike the compounds shown in Chart 1,^11−13^ where the telluroxane spheres are stabilized by a central layer of halide ions, they are stabilized rather by covalent bridges established by phenylseleninate building blocks. Charge compensation is provided by nitrate anions (compounds **1** and **2**) or a mixture of nitrate and [Na_2_(NO_3_)_8_]^6–^ anions (compound **3**).

**Scheme 1:**
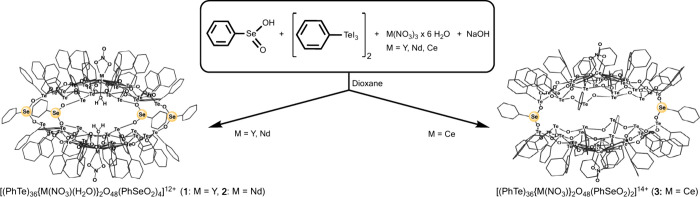
Synthesis of the Chalcogenoxane Clusters (for Experimental Details, See Supporting Information)

Figure 1 depicts the structure of compound **1** with two [(PhTe)_18_{Y(NO_3_)(H_2_O)}O_24_]^8+^ half-spheres connected by four PhSeO_2_^–^ units. These bridges extend the telluroxane clusters of the two subunits to an unprecedented mixed tellurium/selenium [(PhTe)_36_(PhSe)_4_{Y(NO_3_)(H_2_O)}_2_O_56_]^12+^ chalcogenoxane network. Six nitrate anions are located inside the void formed between the half-shells, while the remaining six are situated outside but in the direct neighborhood of the chalcogenoxane cluster.

**Figure 1:**
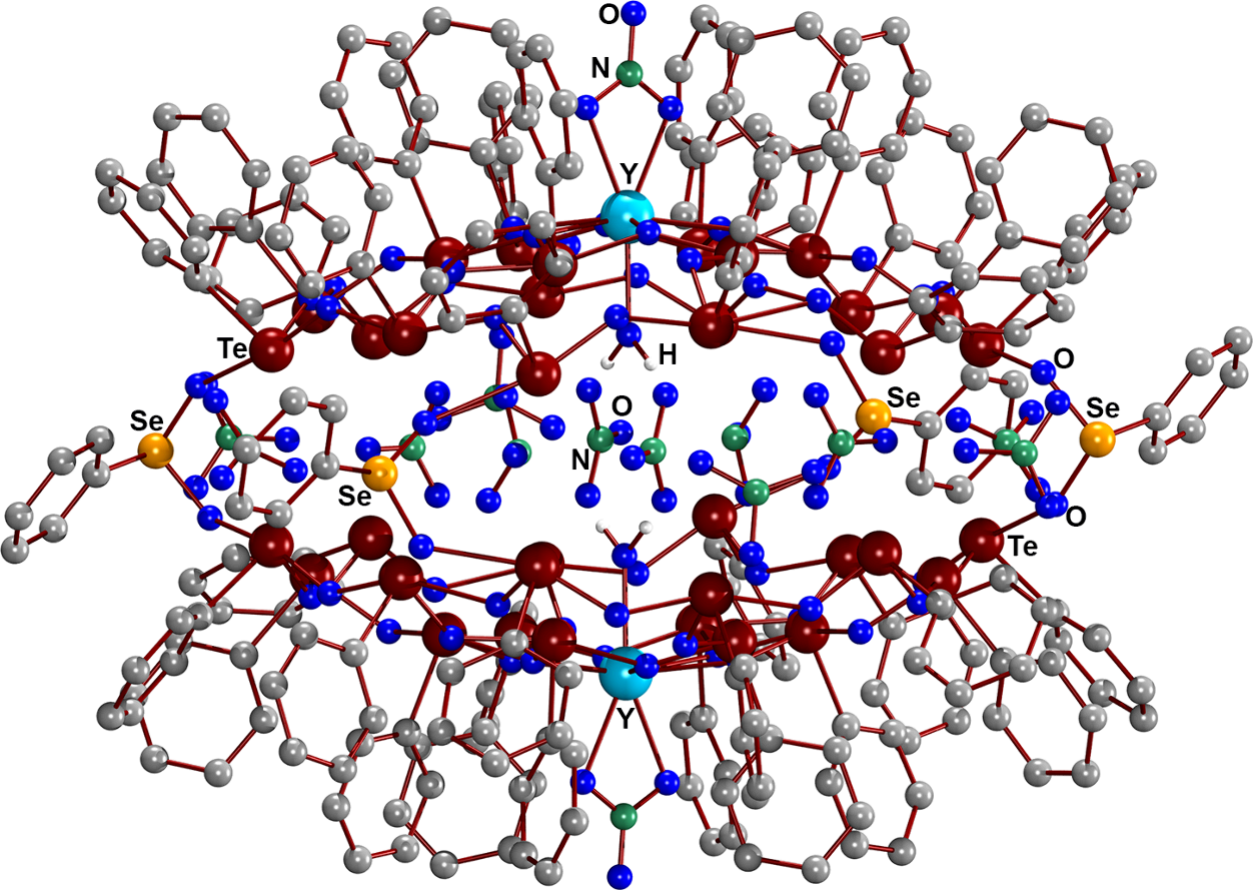
Structure of compound **1**. H atoms are omitted.

While an analogous cluster with four phenylseleninate bridges (compound **2**) is also formed during a reaction with neodymium nitrate, a product with the larger Ce^3+^ ions (compound **3**) shows some structural differences. The two [(PhTe)_18_{Ce(NO_3_)}O_24_]^8+^ telluroxane networks are connected by only two PhSeO_2_^–^ units, and the void between the half-spheres is occupied by a [Na_2_(NO_3_)_8_]^6–^ cluster. Each nitrate in this cluster is shared with the cerium ions of the shell. The remaining nitrate anions are arranged in the direct periphery of the formed sphere. Figures depicting the complete structure of **3** as well as of the central sodium nitrate cluster are given as Supporting Information. There are also reproductions of the “empty” chalcogenoxane skeletons illustrating the large voids between the two half-spheres. These voids account for approximately 1500 Å^3^.

Figure 2 shows top views of the telluroxane networks of **1** and **3** considering Te–O contacts between 1.7 and 3.6 Å. It is evident that these structures are quite similar, independent of the number of the phenylseleninate bridges. The Y–O bonds inside this network range between 2.400(7) and 2.224(7) Å and the Ce–O bonds between 2.506(7) and 2.554(7) Å. This gives an overall bonding situation as observed before for the halide-layered telluroxanes (see compound **4** in Figure 3).^11−13^

**Figure 2:**
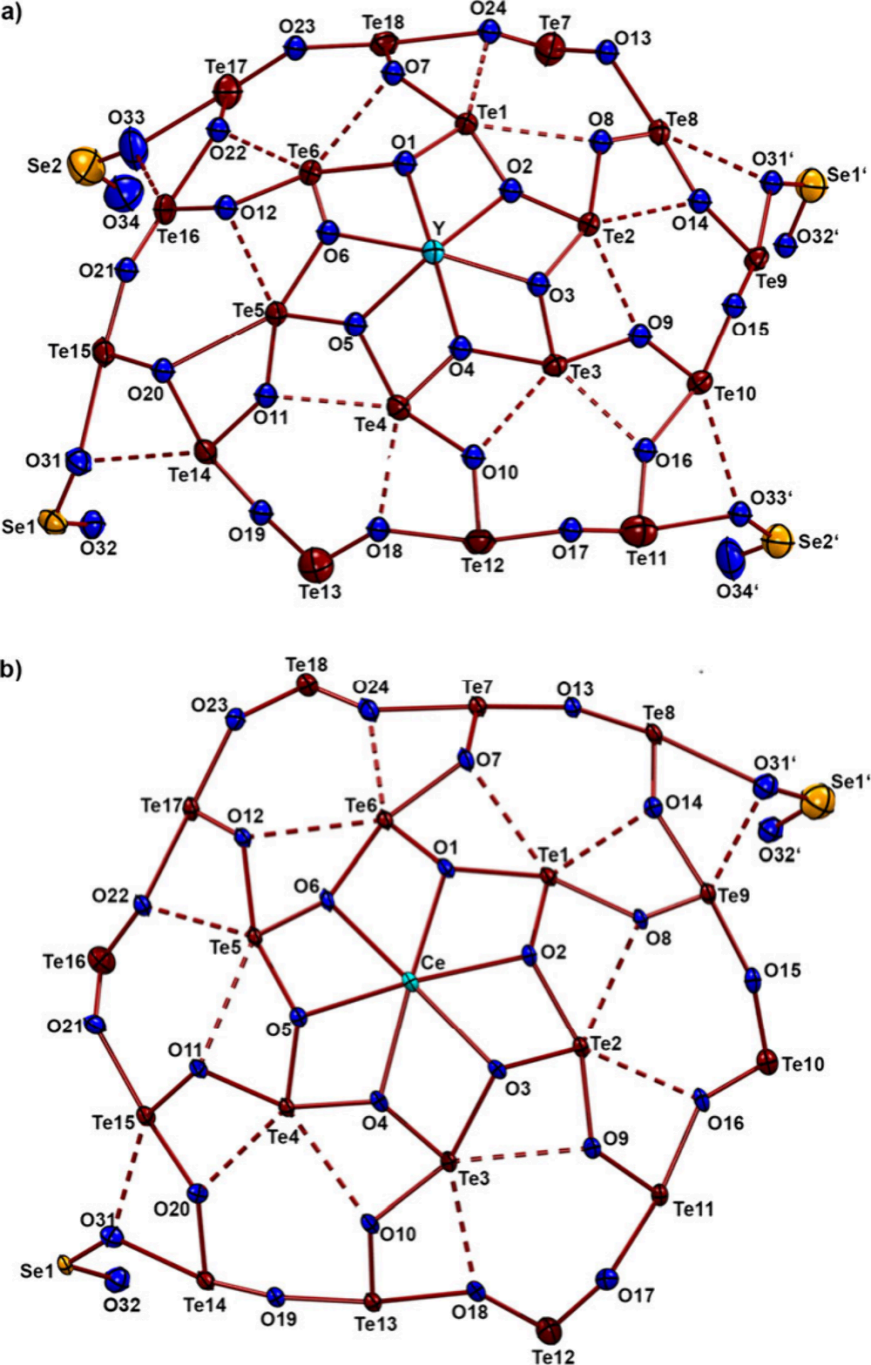
Top-views to the telluroxane networks of compounds **1** and **3**. Solid lines represent Te–O bonds < 2.4 Å, while the dashed lines are contacts between 2.4 and 3.6 Å.

**Figure 3:**
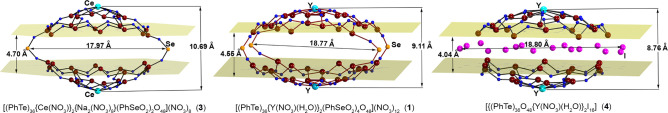
Size and shape of the spherical chalcogenoxane clusters **1** and **3** and their iodine layered analog [{(PhTe)_18_O_24_Y(NO_3_)(H_2_O)}_2_I_16_] (**4**).^13^

The telluroxane networks of the [(PhTe)_18_{ML}O_24_]^7+/8+^ half-spheres seem to be structurally flexible and adopt a concavity, depending on the occupation of the central void. This is illustrated in Figure 3. It becomes clear that the distances of the mean least-squares planes formed by the outer-sphere tellurium atoms (Te7 to Te18 in Figure 2) increase when going from the iodide-layered cluster **4** via compound **1** to complex **3**. The short distance in **4** may be understood by the generally different connection between the half-spheres, while the phenylseleninate-bridged compounds **1** and **3** do not show significant differences in the O–Se–O angles of the bridging units (104–106°). It is more probable that the steric requirements of the central sodium nitrate cluster (for its structure, see the Supporting Information), which also coordinates to the two cerium atoms, are the reason for the larger Ce–Ce distance in **3** compared to the Y–Y one in **1**.

It is interesting to note that compounds **1**–**3** are thermally surprisingly stable, and no difference was found between the compounds with two and four PhSeO_2_^–^ bridges. A preliminary TGA study shows that all three compounds are stable up to approximately 200 °C. Interestingly, no defined release of solvent molecules below this temperature is found, but a steady weight loss of approximately 10% is observed for all compounds. The ongoing decompositions at higher temperatures exhibit features remarkably similar to those of the three cluster compounds. They decompose between 200 and 250 °C under complete release of all organic components (including the PhSeO_2_^–^ building blocks), the remaining solvent molecules, the M^3+^ and Na^+^ ions, as well as the nitrate counterions. The measured weight loss corresponds to the formation of an oxidic material, which consists of a more or less defined mixture of TeO_2_ and elemental tellurium. Such mixtures have occasionally been found to possess a remarkable stability and are sometimes interpreted as “Te(II) oxide.”^14^ This is supported by the detection of consecutive smaller but defined degradation steps at higher temperatures. Unfortunately, the formed products were amorphous and could not be studied crystallographically.

In contrast to the iodide-layered telluroxane clusters (e.g., compound **4**),^13^ which are readily ionized in a mass spectrometer and give spectra with peaks of high intensity at high mass ranges, the spectra of the compounds **1** and **3** show each one peak group only at approximately *m*/*z* = 4900 (**1**) and *m*/*z* = 4850 (**3**). They are given as Supporting Information and belong to the M^2+^ ions. Individual ions can be assigned to species, which contain both half-spheres together with a variable number of phenyl seleninate bridges and nitrate ions. These findings suggest significant contributions of ionic interactions for the stabilization of such clusters, as has been found before for the iodide-connected half-spheres.^13^ For compound **3**, no peaks could be observed in the higher mass range area.

The transfer of more or less intact, ion-filled spheres into the gas phase during the MS experiments and the results obtained from the TGA encouraged us to make a (preliminary) assessment of the acting forces in the clusters under study by means of DFT calculations at the B3LYP level in the gas phase. The results should be suitable to estimate the role of the bridging PhSeO_2_^–^ handles for the stabilization of the dimeric units as an “alternative solution” for the halide layers in the compounds shown in Chart 1d and e.^11−13^ Due to the exorbitant computational cost of theoretical computations related to the large size of the telluroxanes and the assumption that the central metal will only have a minor influence on the energetics of the outer anion sphere, calculations were performed on a model system that has already been computed previously for the all-iodide derivative: the calcium hydrate containing cluster [{(PhTe)_18_O_24_Ca(H_2_O)_2_)}_2_I_16_].^13^ Iodide ions of the outer sphere were gradually replaced by phenylseleninate, and the relative energy differences per PhSeO_2_^–^ unit were then estimated after geometry optimization. The calculations suggest a linear increase in the thermodynamic stability of the compounds by ca. 6–7 kJ/mol per PhSeO_2_^–^ unit. Details are given in the Supporting Information.

The relatively low energy gain per I^–^/PhSeO_2_^–^ replacement suggests that the formation of the phenylseleninate-bridged clusters of the present study in favor of those having the iodide layers is not caused by a potentially covalent character of these bridges. This is supported by the results of the DFT calculations, which characterize the interactions between the PhSeO_2_^–^ and the {PhTeO_*x*_}^+^ units as those between iodide and the {PhTeO_*x*_}^+^ units to be ionic, noncovalent interactions (see Supporting Information). A reduced density gradient, localized orbital locator, electron localization function, and Laplacian mappings clearly visualize the noncovalent character and similarities, while topological features and NBO analyses are consistent with the intuitive interpretation of the graphical representations. Details of the calculations are provided as Supporting Information.

It remains to explore the mechanism of the formation of the cluster compounds and to verify that the exclusive formation of compounds **1** to **3** under the conditions described in this Communication is not only attributed to their low solubility or whether ionic interactions with the ions inside and around the two half-spheres play a major role. The latter questions can potentially be answered by a much more detailed computational study, which also includes the questions of how solvation as well as the filling affect the thermodynamic stability of the clusters. Such further calculations beyond the discussed model system presented preliminarily in the current manuscript are ongoing as of this writing.

## Supporting Information Available

Synthesis details, crystallographic information, vibrational spectra, mass spectra, UV/vis spectra, TGA data, computational details (PDF)
Coordinated for the optimized structures used for the calculations (XYZ)

## Supplementary Material

ic4c02274_si_001.pdf

ic4c02274_si_002.xyz

## References

[ref1] Beckmann J.; Finke P. Organotelluroxanes. In Selenium and Tellurium Chemistry: from Small Molecules to Biomolecules and Materials; Woolins J. S., Laitinen R., Eds.; Springer: Berlin, 2011; pp 151–177.

[ref2] Srivastava K.; Panda A.; Sharma S.; Singh H. B. Telluroxanes: synthesis, structure and applicatios. J. Organomet. Chem. 2018, 861, 174–206. 10.1016/j.jorganchem.2018.02.036.

[ref3] Deka R.; Sarkar A.; Butcher R. J.; Junk P. C.; Turner D. R.; Deacon G. B.; Singh H. B. Isolation of the novel example of a monomeric organotellurinic acid. Dalton Trans. 2020, 49, 1173–1180. 10.1039/C9DT04013G. 31895377

[ref4] Gupta A.; Deka R.; Sarkar A.; Singh H. B.; Butcher R. J. Oxidation behavior of intramolecularly coordinated unsymmetrical diorganotellurides: isolation of novel tetraorganoditelluronic acids, [RR’Te(μ-O)(OH)_2_]_2_. Dalton Trans. 2019, 48, 10979–10985. 10.1039/C9DT01926J. 31210248

[ref5] Beckmann J.; Duthie A.; Gesing T. M.; Koehne T.; Lork E. Depolymerization of Aryltellurinic Anhydrides with Sodium Hydroxide. Synthesis and Structure of the Hydrated Sodium Aryltellurinates [Na(H_2_O)_4_](RTeO_2_) (R = 4-MeOC_6_H_4_, 8-Me_2_NC_10_H_6_). Organometallics 2012, 31, 3451–3454. 10.1021/om300130a.

[ref6] Oba M.; Nishiyama K.; Koguchi S.; Shimada S.; Ando W. Synthesis and Properties of Tellurinic Anhydride–Tellurone Adducts. Organometallics 2013, 32, 6620–6623. 10.1021/om400772d.

[ref7] Beckmann J.; Finke P.; Hesse M.; Wettig B. Well-Defined Stibonic and Tellurinic Acids. Angew. Chem., Int. Ed. 2008, 47, 9982–9984. 10.1002/anie.200803997. 19006136

[ref8] Srivastava K.; Sharma S.; Singh H. B.; Singh U. P.; Butcher R. J. Hydrolysis of 2-phenylazophenyltellurium trihalides: isolation of an unprecedented homometallic, discrete heptanuclear organotellurium oxide cluster. Chem. Commun. 2010, 46, 1130–1132. 10.1039/B921785A. 20126736

[ref9] Beckmann J.; Bolsinger J.; Duthie A. The Reactivity of Diorganotellurium Oxides Towards Phenol and o-Nitrophenol. Hypervalent and Secondary Bonding of Four Different Product Classes. Aust. J. Chem. 2008, 61, 172 10.1071/CH07329.

[ref10] Beckmann J.; Bolsinger J.; Duthie A. Intramolecularly Coordinated Telluroxane Clusters and Polymers. Chem.—Eur. J. 2011, 17, 930–940. 10.1002/chem.201002371. 21226110

[ref11] Kirsten L. Transition metal complexes and cluster compounds starting from functionalized telluroethers. Doctoral thesis, Freie Universität Berlin, 2017. https://refubium.fu-berlin.de/handle/fub188/6152.

[ref12] Yadav S.; Singh H. B.; Zeller M.; Butcher R. J. From Mixed-Valent Phenyltellurenyl Bromide Ph(Br_2_)TeTePh to the Isolation of [{(C_6_H_5_)Te}_19_O_24_Br_5_]Br_4_. Organometallics 2017, 36, 2067–2071. 10.1021/acs.organomet.6b00953.

[ref13] Kirsten L.; Fonseca Rodrigues J.; Hagenbach A.; Springer A.; Pineda N. R.; Piquini P. C.; Roca Jungfer M.; Schulz Lang E.; Abram U. Large Telluroxane Bowls Connected by a Layer of Iodine Ions. Angew. Chem., Int. Ed. 2021, 60, 15517–15523. 10.1002/anie.202103700. PMC8361918 33939866

[ref14] Tyan Y.-S.; Preuss D. R.; Vazan F.; Marino S. J. Laser recording in tellurium suboxide thin films. J. Appl. Phys. 1986, 59, 716–719. 10.1063/1.336588.

