# Supplementary material for: Phenylseleninate-Connected Telluroxane Clusters
Source: Inorg Chem. 2024 Aug 26;63(36):16590–4. doi: 10.1021/acs.inorgchem.4c02274 (PMC11388459; doi:10.1021/acs.inorgchem.4c02274)
Supplement: Supplementary file 1 — ic4c02274_si_001.pdf [file ic4c02274_si_001.pdf]

Supporting Information

Phenylseleninate-Connected Telluroxane Clusters

Jéssica Fonseca Rodrigues,[§] Ana Júlia Zimmermann Londero,[§] Bárbara Tirloni,[§] Maximilian Roca Jungfer,[$] Ulrich Abram*[#] and Ernesto Schulz Lang*[§]

[§] Departamento de Química, Universidade Federal de Santa Maria – UFSM, Laboratório de Materiais Inorgânicos – LMI, 97105-900, Santa Maria, RS, Brazil. E-mail: eslang@ufsm.br

[$] Ruprecht-Karls Universität Heidelberg, Im Neuenheimer Feld 271, D-69120 Heidelberg, Germany

[#] Institute of Chemistry and Biochemistry, Freie Universität Berlin, Fabeckstr. 34-36, D-14195 Berlin, Germany. E-mail: ulrich.abram@fu-berlin.de

Table of Contents

1. **General Considerations** 2
2. **Syntheses** 2
3. **Thermogravimetry (TGA)** 4
4. **X-Ray diffraction** 5
5. **Spectroscopic Data** 8
6. **Computational Details** 13
7. **References** 26

1. General Considerations

Commercially purchased starting materials were used as received without further purifications. Phenyltellurium triiodide ($PhTeI_3$) and phenylseleninic acid ($PhSeO_2H$) were synthesized according to the literature.[1,2] The synthesis of the clusters was carried out using a vacuum-line and Schlenk technique under an argon atmosphere. Elemental analyses were performed on vacuum-dried samples.

Physical Measurements

Elemental analysis (CHNS) was determined with a Perkin-Elmer CHN 2400 elemental analyzer. Mass spectrometric experiments (ESI-MS) have been carried out with $CHCl_3$ solutions on a Synapt G2-S HDMS system equipped with a Z-Spray ESI source (Waters Co., Milford, MA, USA). UV-vis spectra were measured in $CHCl_3$ on a UV-vis 1650-PC Shimadzu spectrometer in the wavelength range between 250 and 800 nm and a data interval of 0.2 nm. The melting points were determined on a Microquímica MQAPF-301 melting point apparatus and are uncorrected. FT-IR spectra were measured using a Bruker LUMOS spectrometer in the wavenumber range of 600–4000 cm^{-1}. Confocal Raman spectra (3600–50 cm^{-1}) were recorded on a Bruker Senterra micro-Raman spectrometer using a 785 nm laser line (diode laser), which was focused onto the samples by a 20x Olympus objective (NA 0.40). Thermogravimetric analyses (TGA) were performed using a TGA Q500 (TA Instrument) at a heating rate of 1 °C min^{-1}, under a continuous flow of nitrogen gas (rate 45 mL min^{-1}) in the temperature range of 1–950 °C.

Single-crystal data were collected with a Bruker D8 Venture diffractometer operating with an Incoatec X-ray source with Montel two-dimensional optics, Mo-Kα radiation (λ = 0.71073 Å), and a Photon 100 detector. The structures were solved by dual space methods using Bruker XT and refined with Bruker XL on F^2, using anisotropic temperature parameters for the non-hydrogen atoms.[3-5] The positions of the hydrogen atoms were calculated for idealized positions. Crystal data and more details regarding the data collection and refinement of compounds are discussed in a separate section. CCDC 2172185 and CCDC 2337905 contain the supplementary crystallographic data for compounds **1** and **3**. These data can be obtained free of charge at http://www.ccdc.cam.ac.uk, from the Cambridge Crystallographic Data Centre, at 12 Union Road, Cambridge CB2 1EZ, UK; *via* fax: (+44) 1223-336-033; or *via* e-mail: deposit@ccdc.cam.ac.uk).

2. Syntheses

$PhTeI_3$ (58.5 mg, 0.1 mmol) and 0.1 mmol of $M(NO_3)_3 \cdot 6\ H_2O$ salts (M = Y: 38 mg, M = Nd: 44 mg, M = Ce: 43 mg) were suspended in 8 mL 1,4-dioxane under an atmosphere of argon. A brick-red solid precipitated within 2 h of stirring at room temperature. The addition of $PhSeO_2H$ (0.2 mmol, 30 mg) in 4 mL of 1,4-dioxane and a few drops of dilute NaOH) resulted in the formation of a clear, brownish solution within 4 min. The stirring was continued for one more hour and the reaction mixture was filtered through Celite. After storing in a desiccator for 4 days, golden prismatic crystals deposited from the orange-red solution formed. They were isolated by filtration

and directly used for the X-ray diffraction experiments. The $PhTeI_3$/$Ln(NO_3)_3$/$PhSeO_2H$ ratio described gave optimal yields for all three products. The use of variable ratios of the reactants did yield not different products, particularly no change in the number of $PhSeO_2^-$ bridges was observed.

[{$(PhTe)_{18}${Y(NO_3)(H_2O)}O_{24}}$_2$($PhSeO_2$)$_4$(NO_3)$_{12}$] (1): Yield: 50% based on $PhTeI_3$. Golden-yellow crystalline solid. Melting point: decomposition initiates between 231 and 233°C. Elemental analysis calcd for $C_{240}H_{204}N_{14}O_{100}Se_4Te_{36}Y_2$: C, 28.91; H, 2.06; N, 1.97%. Found: C 28.4; H, 1.69; N, 1.93%. IR (ATR, cm^{-1}): 3053 (w), 1518 (w), 1477 (m), 1435 (m), 1282 (s), 1060 (m), 1020 (m), 734 (s). Raman (cm^{-1}): 1480 (w), 1183 (w), 1157 (w), 1062 (w), 1041 (m),1021 (m), 999 (s), 834 (w), 662 (m), 613 (w), 485 (w), 432 (w), 255 (m). UV-Vis ($CHCl_3$): 275 nm, 435 nm, 463 nm. Reliable extinction coefficients could not be determined due to the low solubility of the compound.

[{$(PhTe)_{18}${Nd(NO_3)(H_2O)}O_{24}}$_2$($PhSeO_2$)$_4$(NO_3)$_{12}$] (2): Yield: 28% based on $PhTeI_3$. Golden-yellow crystals, which slowly decompose upon standing in air (most probably by the loss of incorporated solvent). Melting point: decomposition initiates between 230 and 233°C. Elemental analysis calcd for $C_{240}H_{204}N_{14}O_{100}Se_4Te_{36}Nd_2$: C, 28.59; H, 2.04; N, 1.94%. Found: C, 30.24; H, 2.15; N, 1.94%. The deviations of the carbon (and hydrogen) values are most probably due to residual, co-crystallized dioxane, which could not be completely removed in vacuum at elevated temperatures. Heating resulted in gradual decomposition of the sample. FT-IR (ATR, cm^{-1}): 3053 (m), 2853 (w), 1505 (w), 1478 (m), 1436 (m), 1282 (s), 1060 (m), 1020 (m), 998 (m), 734 (s), 666 (vs). Raman (cm^{-1}): 1576 (m),1480 (w), 1186 (w), 1158 (w), 1058 (w), 1041 (m),1021 (m), 999 (s), 789 (w), 713 (m), 662 (m), 613 (w), 476 (m), 433 (m), 299 (w), 255 (m). UV-Vis ($CHCl_3$): 273 nm, 435 nm, 463 nm. Reliable extinction coefficients could not be determined due to the low solubility of the compound.

[{$(PhTe)_{18}${Ce$(NO_3)_2$}O_{24}}$_2$($PhSeO_2$)$_2${$Na_2(NO_3)_6$}(NO_3)$_8$] (3): Yield: 25% based on $PhTeI_3$. Golden-yellow crystals, which slowly decompose upon standing in air (most probably by the loss of incorporated solvent). Melting point: decomposition initiates between 228 and 230°C. Elemental analysis calcd for $C_{228}H_{190}Ce_2N_{18}Na_2O_{106}Se_2Te_{36}$: C, 27.51; H, 1.92; N, 2.53%. Found: C, 28.76; H, 2.28; N, 2.40%. FT-IR (ATR, cm^{-1}): 3051 (w), 1503 (w), 1478 (w), 1436 (m), 1334 (s), 1301 (s), 1183 (w), 1157 (w), 1119 (m), 1081 (w), 1060 (m), 1037 (w), 872 (w), 817 (w), 734 (vs), 665 (vs). Raman (cm^{-1}): 1575 (m), 1478 (w), 1435 (w), 1185 (w), 1157 (w), 1057 (w), 1019 (m), 998 (s), 736 (m, broad), 704 (m, broad), 660 (m), 613 (m, broad), 465 (m, broad), 425 (m, broad), 250 (s), 159 (s). UV-Vis ($CHCl_3$): 273 nm, 435 nm, 463 nm. Reliable extinction coefficients could not be determined due to the low solubility of the compound.

3. Thermogravimetry (TGA)

In order to estimate the amount of co-crystallized solvent 1,4-dioxane and to study the decomposition of the compounds, the thermal behaviour of crystalline samples of pure crystalline samples of the three clusters were studied by TGA analyses. The results are depicted in Figure S1 showing the mass variation (green curves) and its first derivative (blue curves). The analysis was carried out under a nitrogen atmosphere by heating the samples in a platinum pan, in the range 1 - 950°C, and a rate of 1 deg min^{-1}.

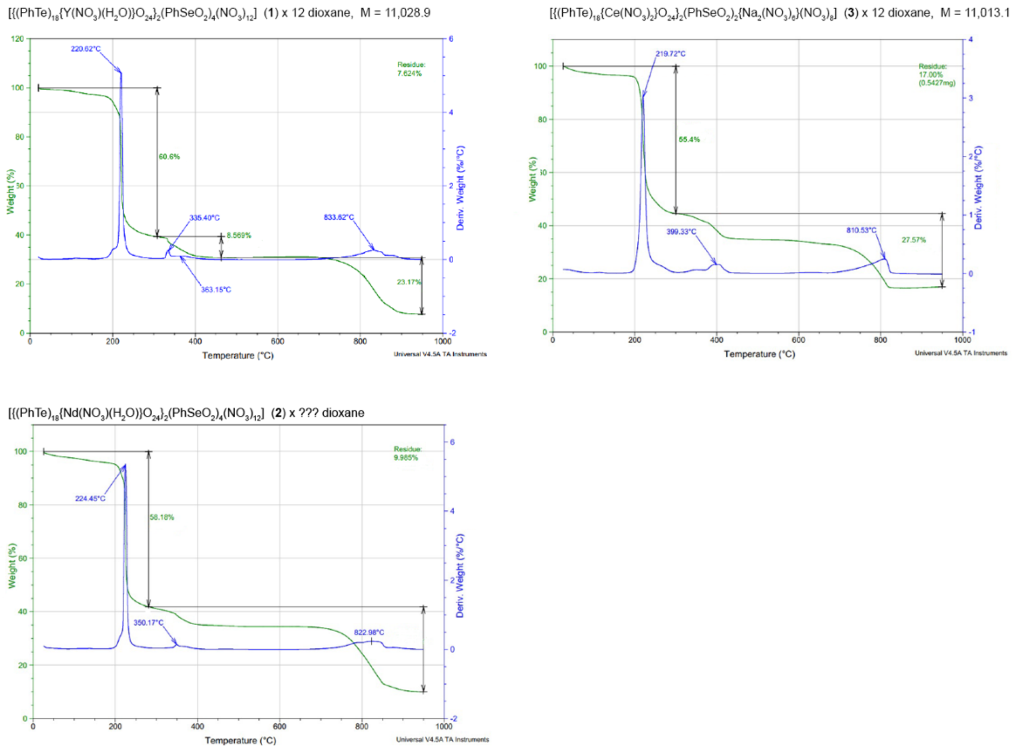

Figure S1. TGA graphs of compounds **1** – **3**.

All three compounds are essentially stable up to approximately 200°C. Interestingly, no defined release of the solvent molecules below this temperature could be found, but a continuous weight loss. The observed weight loss is in approximate agreement with the findings of the crystallographic studies, where approximately twelve and sixteen equivalents of dioxane have been assigned for compounds **1** and **3**, respectively, based on partially resolved molecules and the final electron counts in the solvent-accessible areas by a solvent mask in OLEX2).[5]

The ongoing decompositions at higher temperatures show remarkably similar features for the three cluster compounds. They decompose between 200 and 250°C under complete release of all organic components (including the $PhSeO_2^-$ building blocks), the remaining solvent molecules, the M^{3+} and Na^+ ions as well as the nitrate counter ions. The measured weight loss corresponds to the formation of an oxidic material, which consists of a more or less defined mixture of TeO_2 and elemental tellurium. Such mixtures have occasionally been found to possess a remarkable stability and are sometimes interpreted as 'Te(II) oxide'.[6] This is supported by the detection of consecutive smaller, but defined degradation steps at higher temperatures. Unfortunately, the

formed products were amorphous and could not be studied crystallographically. Thus, at the present stage details about the individual products of the thermal degradation cannot be derived from the existing data.

4. X-Ray diffraction

Some problems were found during the integration of data and refinements of structures, which do not occur in small molecules. RIGU and SIMU commands were applied during the refinements. A summary of the crystal data and the refinement parameters is provided in Table S1.

Table S1. Crystal data and structure determination parameters.

	$[\{(PhTe)_{18}\{Y(NO_3)(H_2O)\}O_{24}\}_2$-$(PhSeO_2)_4(NO_3)_{12}]$ (**1**)*	$[\{(PhTe)_{18}\{Ce(NO_3)_2\}O_{24}\}_2(PhSeO_2)_2$-$\{Na_2(NO_3)_6\}(NO_3)_8]$ (**3**)*
Empirical formula	$C_{288}H_{300}N_{14}O_{124}Se_4Te_{36}Y_2$	$C_{228}H_{190}Ce_2N_{18}Na_2O_{106}Se_2Te_{36}$
Formula weight	11028.66	9955.71
Temperature/K	100.0	100.0
Crystal system	triclinic	triclinic
Space group	P-1	P-1
a/Å	21.8491(8)	22.421(6)
b/Å	23.2504(8)	23.401(6)
c/Å	23.5084(9)	25.046(6)
α/°	96.7800(10)	95.284(13)
β/°	116.8740(10)	113.072(13)
γ/°	113.2340(10)	118.003(19)
Volume/Å^3	9126.3(6)	10039(5)
Z	1	1
ρ_{calc} g/cm^3	2.007	2.647
μ/mm^{-1}	3.623	3.038
F(000)	5204.0	4610.0
Crystal size/mm^3	0.236 × 0.187 × 0.147	0.189 × 0.174 × 0.127
Radiation	MoKα (λ = 0.71073)	MoKα (λ = 0.71073)
2Θ range for data collection/°	3.86 to 49.462	3.848 to 49.73
Index ranges	-25 ≤ h ≤ 25, -27 ≤ k ≤ 27, -27 ≤ l ≤ 27	-26 ≤ h ≤ 26, -27 ≤ k ≤ 27, -29 ≤ l ≤ 29
Reflections collected	97517	106813
Independent reflections	30930 [R_{int} = 0.0282, R_{sigma} = 0.0346]	34649 [R_{int} = 0.0435, R_{sigma} = 0.0516]
Data/restraints/parameters	30930/4536/1802	34649/1728/1695
Goodness-of-fit on F^2	1.053	1.056
Final R indexes [I>=2σ (I)]	R_1 = 0.0720, wR_2 = 0.2134	R_1 = 0.0633, wR_2 = 0.1760
Final R indexes [all data]	R_1 = 0.0897, wR_2 = 0.2319	R_1 = 0.0804, wR_2 = 0.1818
Largest diff. peak/hole / e Å^{-3}	4.12/-3.52	3.64/-3.88
CCCD deposit	2172185	2337905

* Solvent masks were calculated, and 552 electrons were found in a volume of 2542 A^3 in one void per unit cell for compound **1** and 733 electrons were found in a volume of 3858 A^3 in one void per unit cell for compound **3**. These values are consistent with the presence of 12 molecules of solvent dioxane in compound **1** and 16 molecules dioxane in compound **3.**

A number of restraints has been applied to model several disordered phenyl rings and nitrate ions in both compounds. ISOR instructions have been used for the treatment of unusual thermal ellipsoids of carbon and oxygen atoms in compound **1**. Ellipsoid representations of the resulting asymmetric units are shown in Fig. S2.

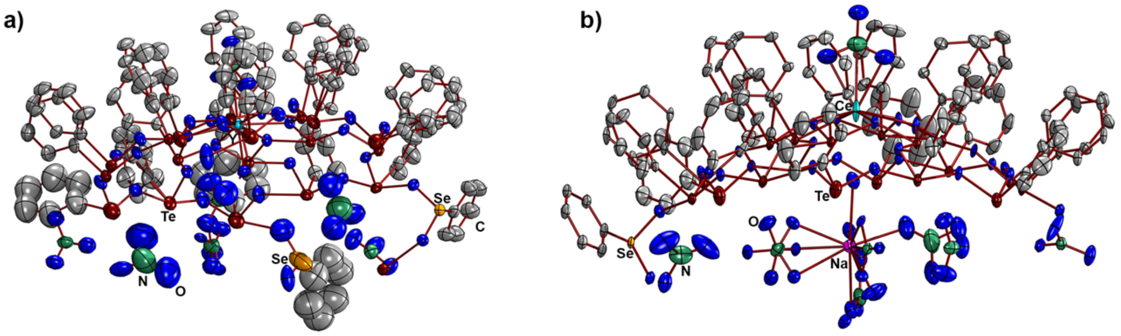

Figure S2. Ellipsoid representations of the asymmetric units of compounds **1** and **3** including the ISOR-refined C atoms and disordered nitrate anions and phenyl rings. Ellipsoids are depicted at mainly 30% probability. Hydrogen atoms were omitted for clarity.

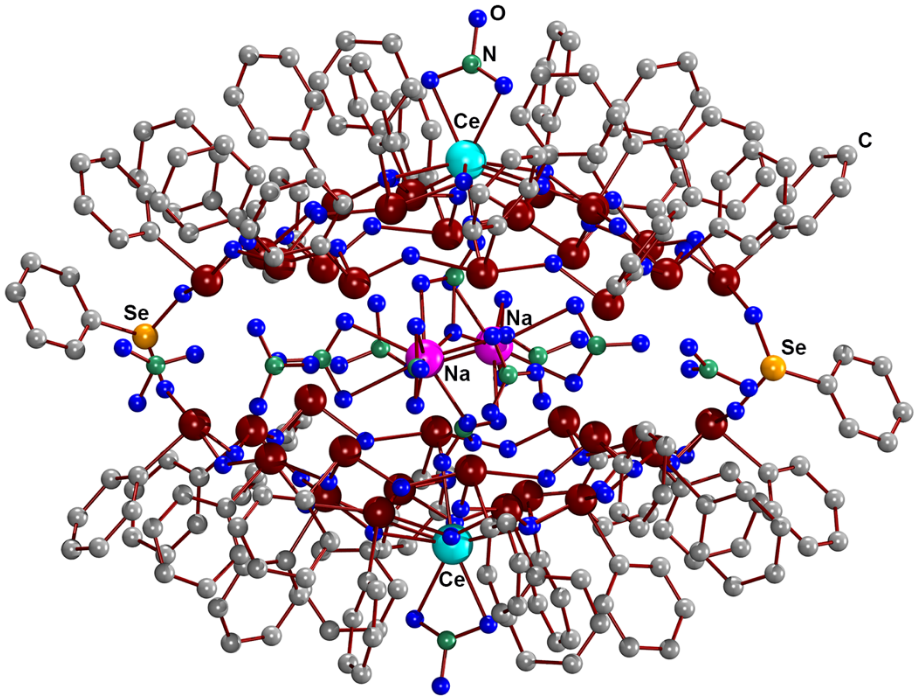

Figure S3. Representation of the complete structure of compound **3** Hydrogen atoms were omitted for clarity.

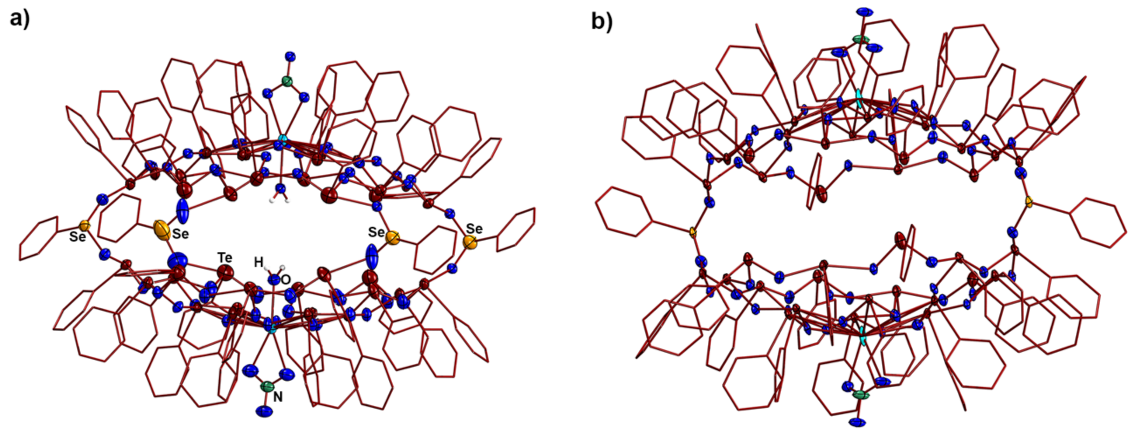

Figure S4. Representations of the empty cations of compounds **1** and **3**. Nitrate and $[Na_2(NO_3)_8]^{6-}$ anions have been removed for clarity.

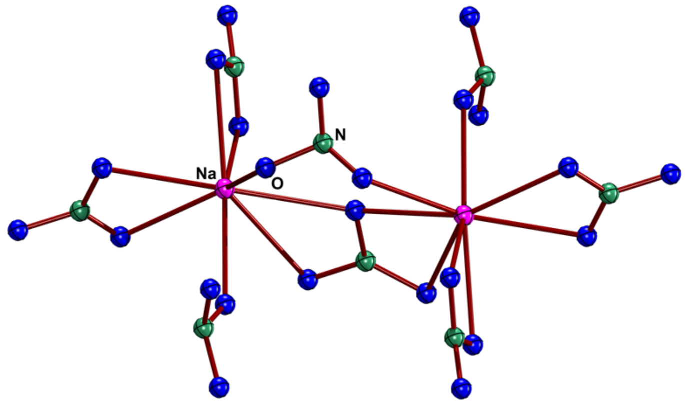

Figure S5. Representation of the $[Na_2(NO_3)_8]^{6-}$ cluster in the centre of compound **3**.

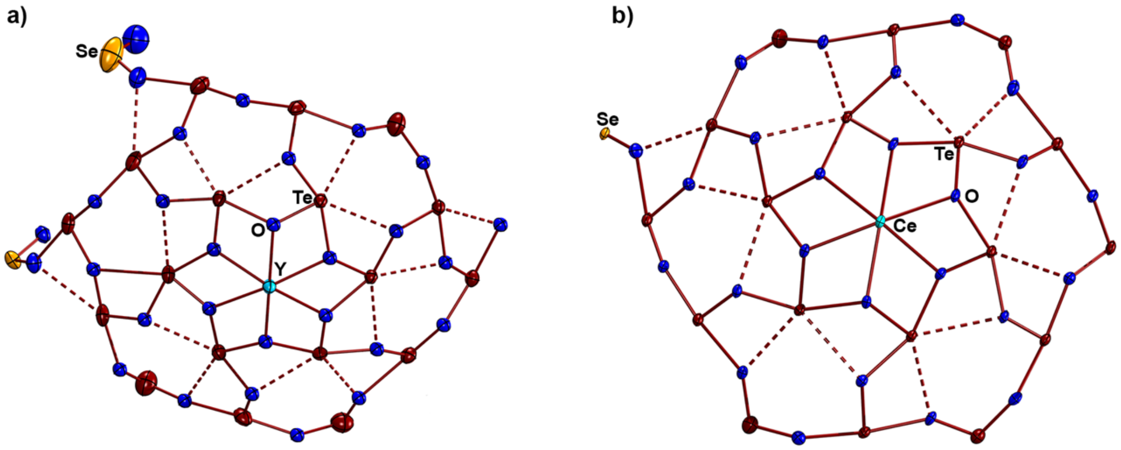

Figure S6. Top view to the tellurium oxide networks with the central Y^{3+} and Ce^{3+} ions of compounds **1** and **3**. Solid lines represent Te-O distances between 1.83 and 2.4 Å, dashed lines such between 2.4 and to 3.6 Å (sum of the van der Waals radii).

5. Spectroscopic Data

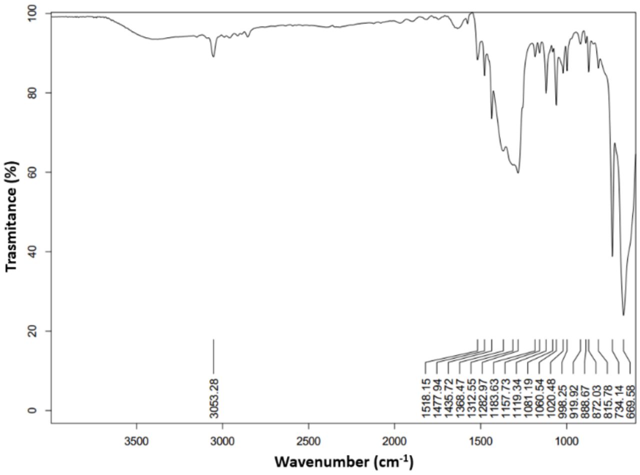

Figure S7. FTIR spectrum of compound **1**.

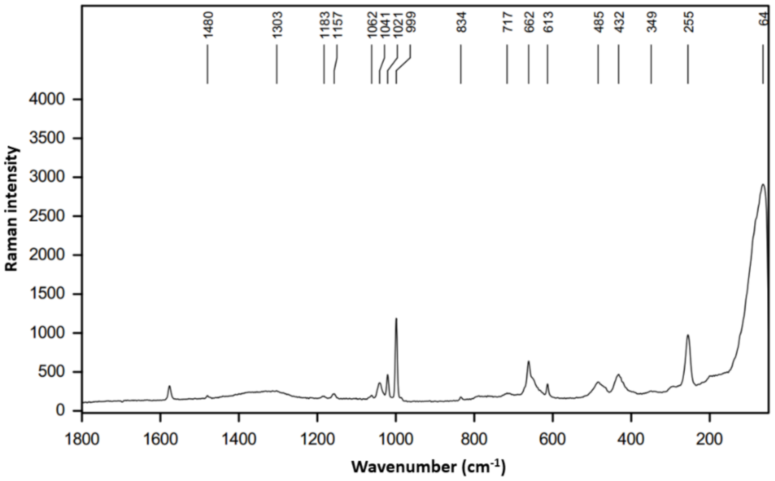

Figure S8. Raman spectrum of compound **1**.

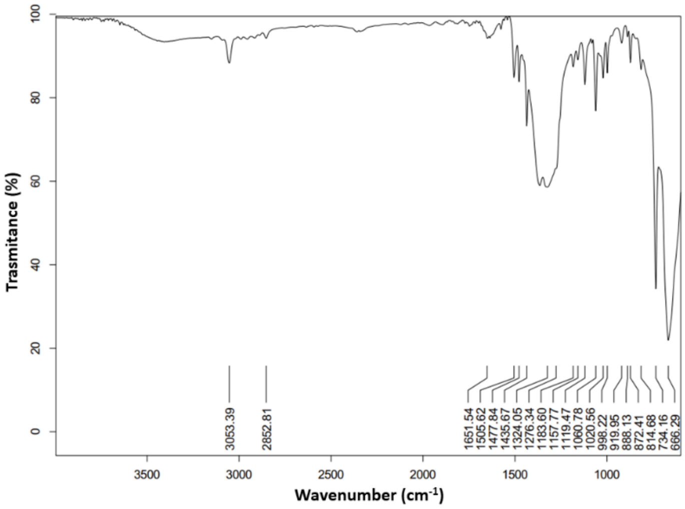

Figure S9. FTIR spectrum of compound **2**.

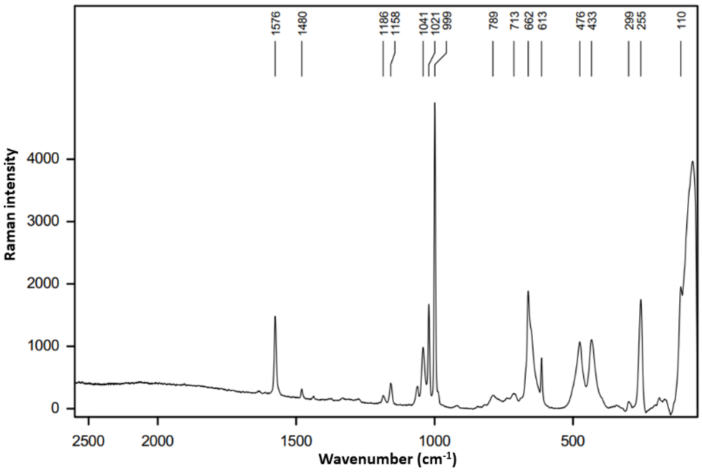

Figure S10. Raman spectrum of compound **2**.

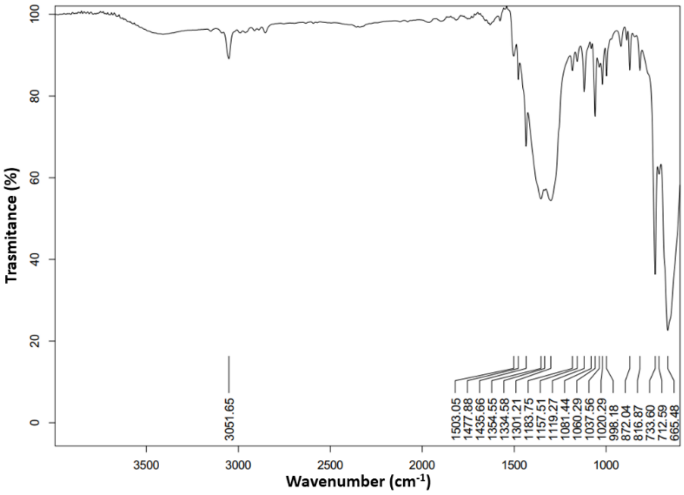

Figure S11. FTIR spectrum of compound **3**.

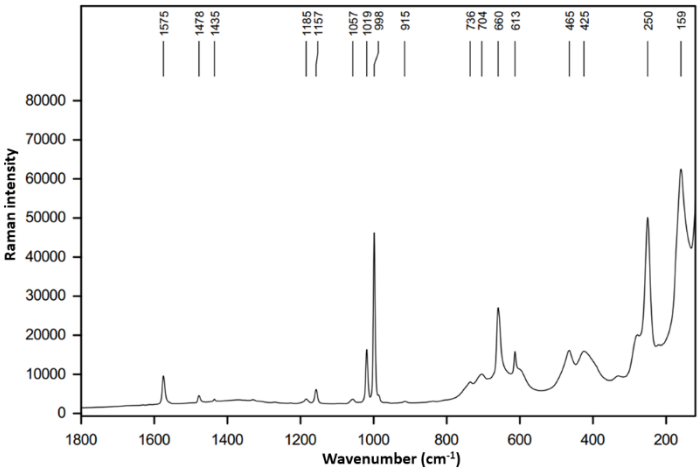

Figure S12. Raman spectrum of compound **3**.

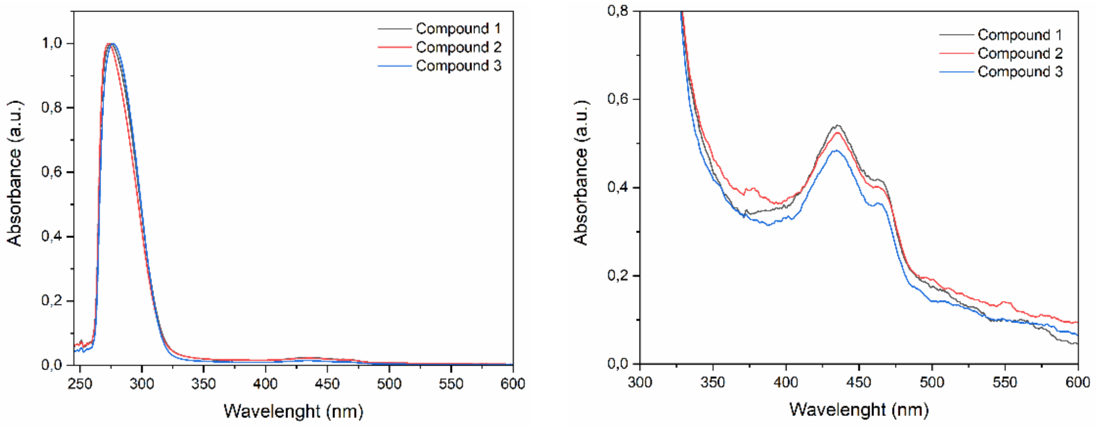

Figure S13. UV-Vis spectra of compounds **1**–**3** in $CHCl_3$. The absorptions around 450 nm can be assigned to charge-transfer excitations (HOMO: anion-based, LUMO telluroxane-based), while the intense excitations at 260 nm should be assigned to π-π* transitions of the cluster skeleton (including the aromatic system). Such an assignment is in accord with previous DFT calculations on analogous iodide-layered systems (*Angew. Chem. Int. Ed.* **2021**, *60*, 15517-15523).[8]

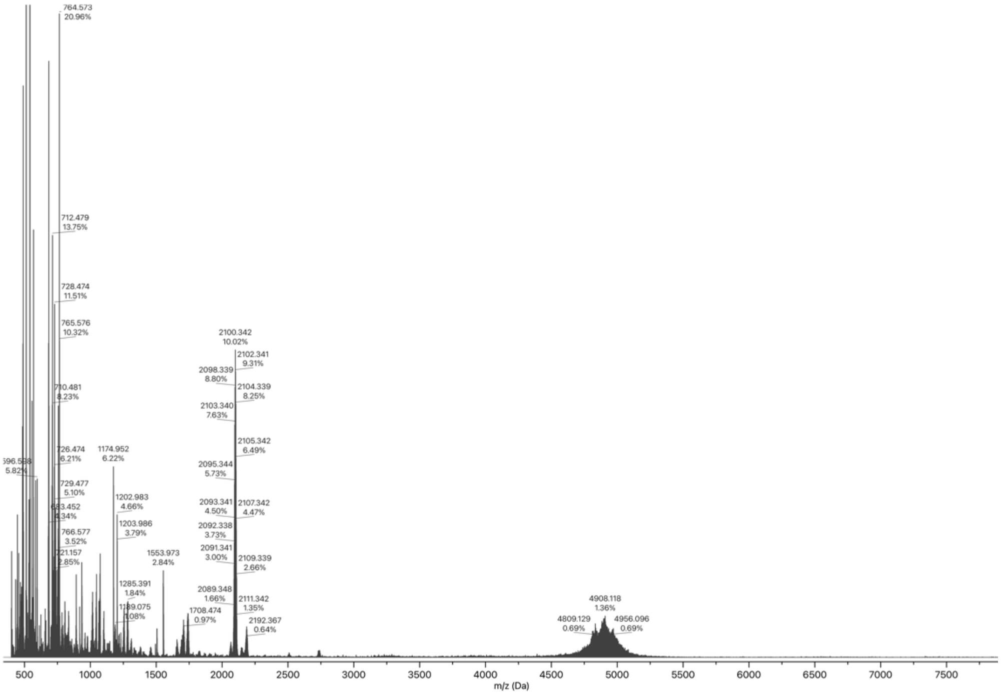

Figure S14. Overview ESI+ mass spectrum of compound **1** in $CHCl_3$.

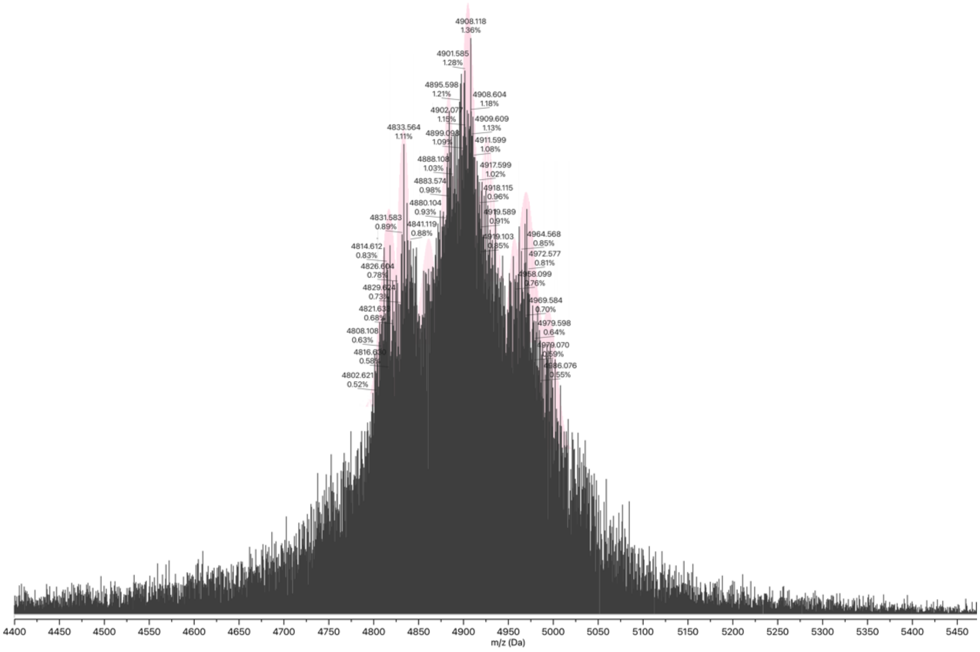

Figure S15. ESI+ mass spectrum of the $[M]^{2+}$ region of compound **1** in $CHCl_3$ together with simulated peaks. The simulated (red) peaks include ions of the compositions $[M\ -PhSeO_2^-, -2\ NO_3^-, -2\ H_2O, +Na^+]^{2+}$, $[M\ -PhSeO_2^-, -2\ NO_3^-, -2\ H_2O, +K^+]^{2+}$, $[M\ -PhSeO_2^-, -NO_3^-]^{2+}$, $[M\ -PhSeO_2^-, -H_2O, +K^+]^{2+}$, $[M\ -2\ NO_3^-, -2\ H_2O]^{2+}$, $[M\ -2\ NO_3^-]^{2+}$, $[M\ -NO_3^-, -H_2O, +Na^+]^{2+}$, $[M\ -NO_3^-, -H_2O, +K^+]^{2+}$, $[M\ -NO_3^-, +K^+]^{2+}$.

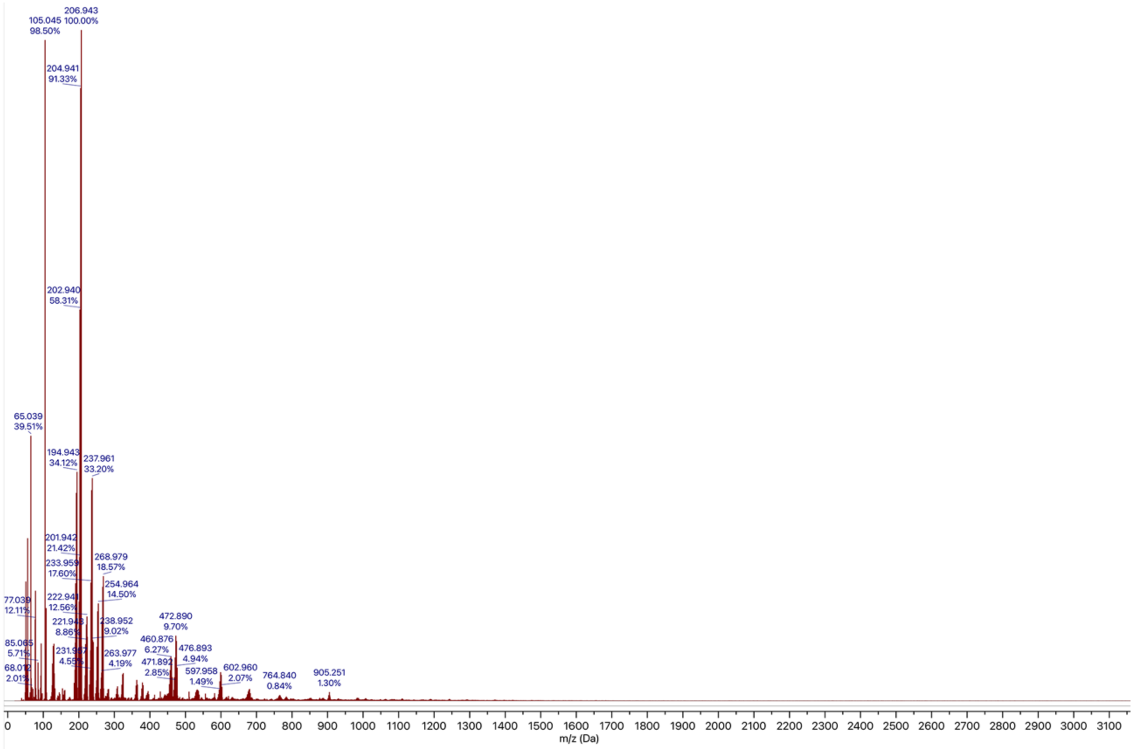

Figure S16. Overview ESI+ mass spectrum of compound **2** in $CHCl_3$.

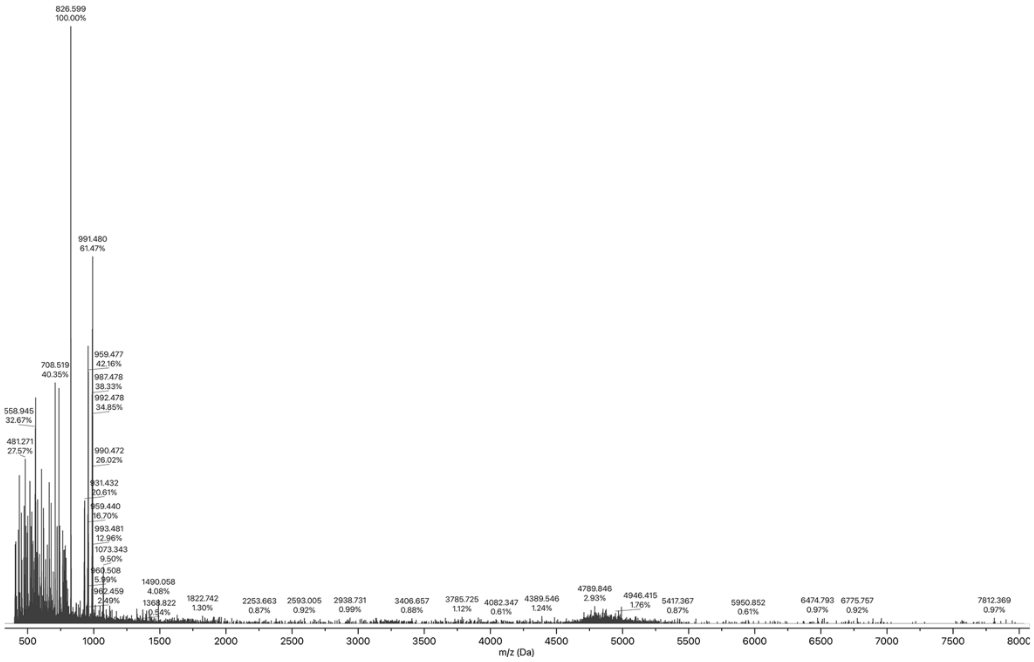

Figure S17. Overview ESI+ mass spectrum of compound **3** in $CHCl_3$.

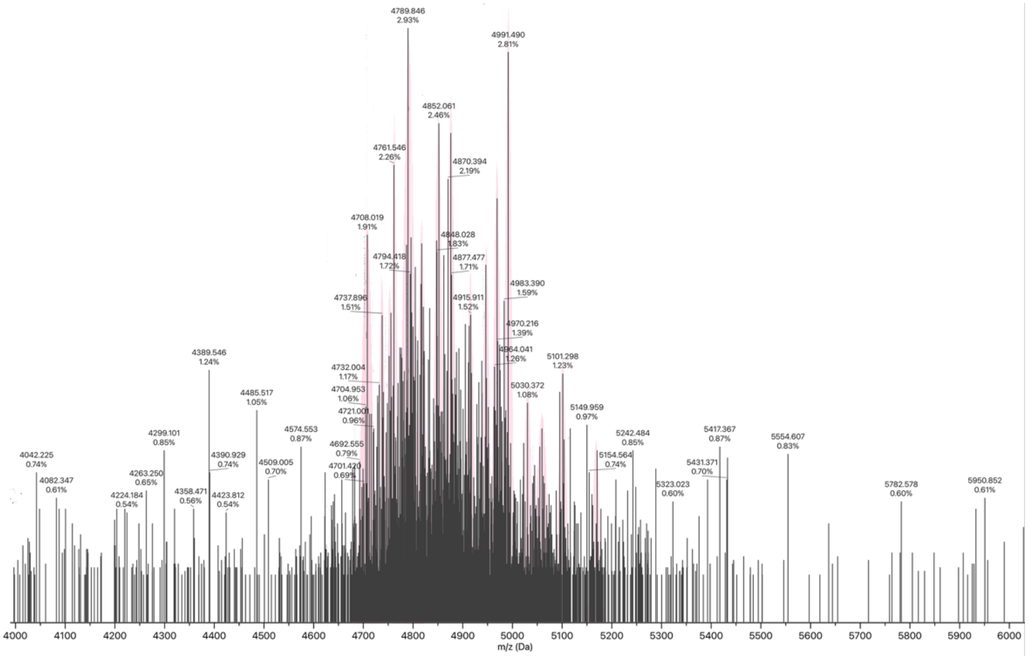

Figure S18. ESI+ mass spectrum of the $[M]^{2+}$ region of compound **3** in $CHCl_3$ together with simulated peaks. The simulated (red) peaks include ions of the compositions [M $-PhSeO_2^-$, $-NO_3^-$, $+H_2O$, -2 Na^+, +2 $K^+]^{2+}$, [M -2 $PhSeO_2^-$, $-NO_3^-$, -2 Na^+, $+H^+]^{2+}$, [M -2 $PhSeO_2^-$, -2 Na^+, $-NO_3^-$, $+H^+]^{2+}$, [M -2 $PhSeO_2^-$, -2 NO_3^-, +2 $Na^+]^{2+}$, [M -2 $PhSeO_2^-$, $-NO_3^-$, $-Na^+]^{2+}$, [M -2PhSeO2-, -2 Na^+, +2 $K^+]^{2+}$, [M $-PhSeO_2^-$, $-NO_3^-]^{2+}$, [M $-NO_3^-$, +2 Cs^+, $+K^+]^{2+}$, [M $-Na^+$, +3 $K^+]^{2+}$, [M $-Na^+$, +3 $Cs^+]^{2+}$, [M +2 $Na^+]^{2+}$, [M $-Na^+$, $+Cs^+]^{2+}$, [M +2 $Cs^+]^{2+}$, [M +2 $K^+]^{2+}$, [M -2 $PhSeO_2^-]^{2+}$, [M -2 $NO_3^-]^{2+}$, [M -2 $PhSeO_2^-$, -2 Na^+, -2 $NO_3^-]^{2+}$.

6. Computational Details

The DFT calculations were performed using Gaussian 16.[7] The initial geometries for the optimizations were derived from the previously optimized structure of [{$(PhTe)_{18}O_{24}Ca$-$(H_2O)_2$}$_2I_{16}$][8] using GaussView.[9] The obtained geometries were reoptimized until the absence of imaginary frequencies verified a true energetic minimum. The light atoms C and H were modelled by simple 3-21G basis functions,[10] while for O, Ca, Se and I LANL2DZ was used.[11,12] For Te the even larger LANL2DZdp was employed.[12-14] For Ca, Se, I and Te, the corresponding effective core potential was used due to the excessive number of atoms involved in each computation. The basis sets were obtained from the Basis Set Exchange database.[15-17] Further analyses of the obtained wave-functions was performed with the free multifunctional wavefunction analyzer Multiwfn.[18] The electron localization function, Laplacian, localized orbital locator and reduced density gradient (RDG)[19] mappings were generated as implemented in Multiwfn with a grid spacing of 2000 x 2000 points. Energies are given in atomic units (a.u.) or kJ/mol as indicated. The optimized structures are shown in Figures S19-S28, while the general idea behind the step-wise substitution is given in visualized in Figure S29 as a schematic cut through the xy- (or iodide) plane. The relative energies are compared in Table S2, while the results of NBO analysis indicating a reasonably similar overall interaction energy between iodide and tellurium compared to phenyl seleninate and tellurium are given in Table S3, while comparable descriptors for the all-iodide reference [{$(PhTe)_{18}O_{24}Ca(H_2O)_2$}$_2I_{16}$] are given in Table S4. Figures S30-S33 show the electron localization function and the localized orbital locator mappings (emphasizing covalent bonds and lone-pairs) as well as the reduced density gradient map (emphasizing the quantum theory of atoms in molecules boundaries of the atoms) and the reduced density gradient map (emphasizing non-covalent interactions and van der Waals contacts) together with the topological critical points of the electron density exemplarily for one of the Se-O···Te moieties. Similarly, Figures S34-S37 show the same maps for a Te···I···Te moiety. Figures S38 and S39 highlight the similarities between the non-covalent interactions and van der Waals contacts in [{$(PhTe)_{18}O_{24}Ca$-$(H_2O)_2$}$_2I_{15}(PhSeO_2)$] and [{$(PhTe)_{18}O_{24}Ca(H_2O)_2$}$_2I_{16}$] based on reduced density gradient maps.

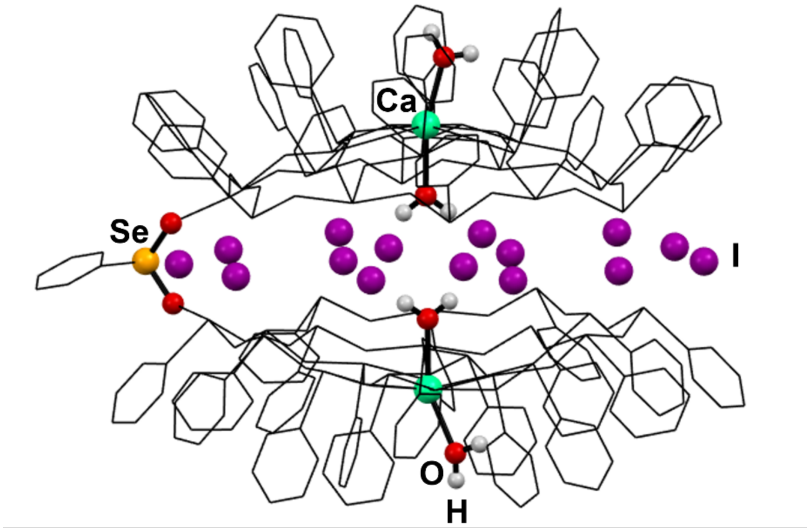

Figure S19. Side-view of the gas-phase optimized structure of [{$(PhTe)_{18}O_{24}Ca$-$(H_2O)_2$}$_2I_{15}(PhSeO_2)$].

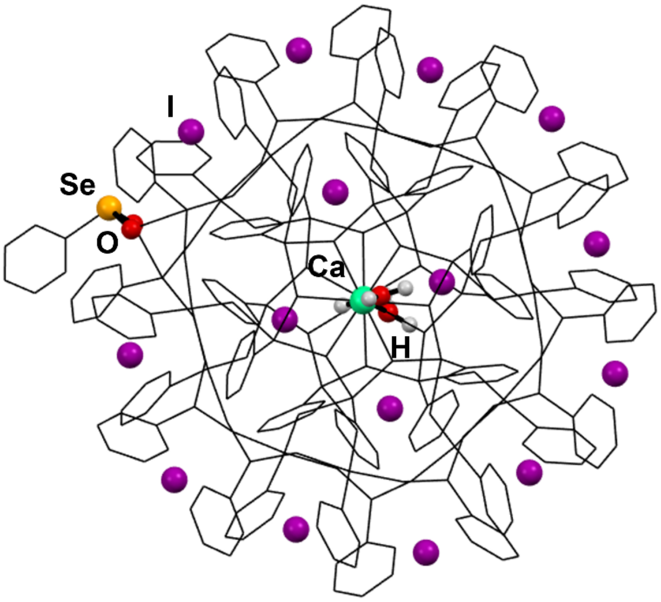

Figure S20. Top-view of the gas-phase optimized structure of [{$(PhTe)_{18}O_{24}Ca$-$(H_2O)_2$}$_2I_{15}(PhSeO_2)$].

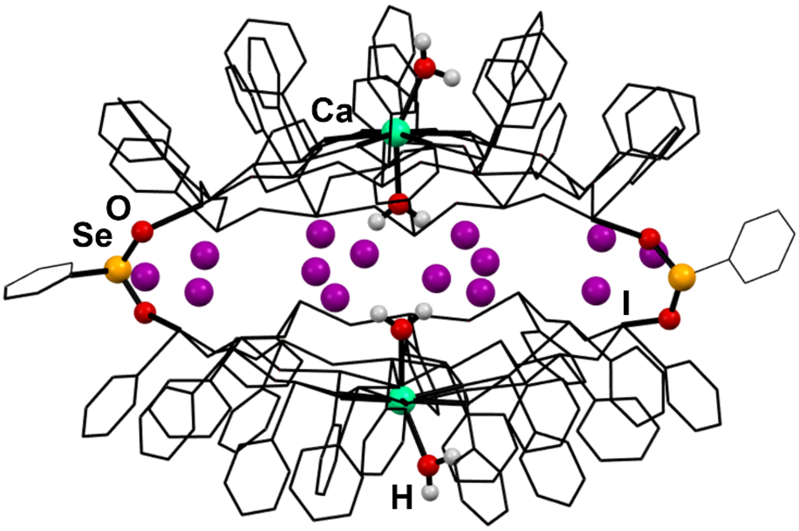

Figure S21. Side-view of the gas-phase optimized structure of [{$(PhTe)_{18}O_{24}Ca(H_2O)_2$}$_2I_{14}(PhSeO_2)_2$].

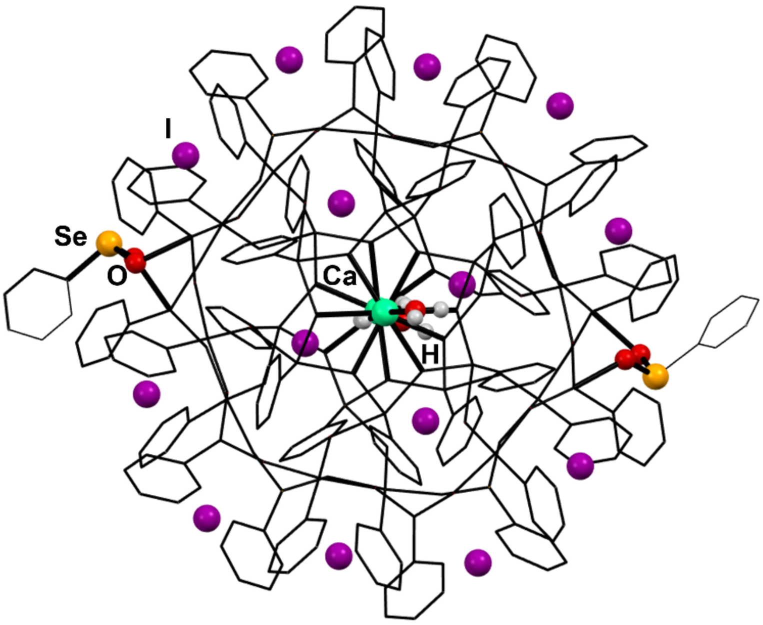

Figure S22. Top-view of the gas-phase optimized structure of [{$(PhTe)_{18}O_{24}Ca(H_2O)_2$}$_2I_{14}(PhSeO_2)_2$].

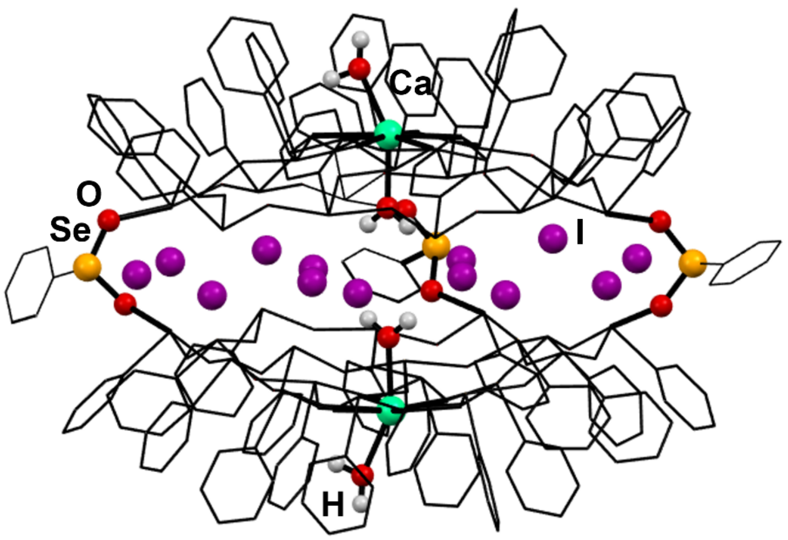

Figure S23. Side-view of the gas-phase optimized structure of $[\{(PhTe)_{18}O_{24}Ca(H_2O)_2)\}_2I_{13}(PhSeO_2)_3]$.

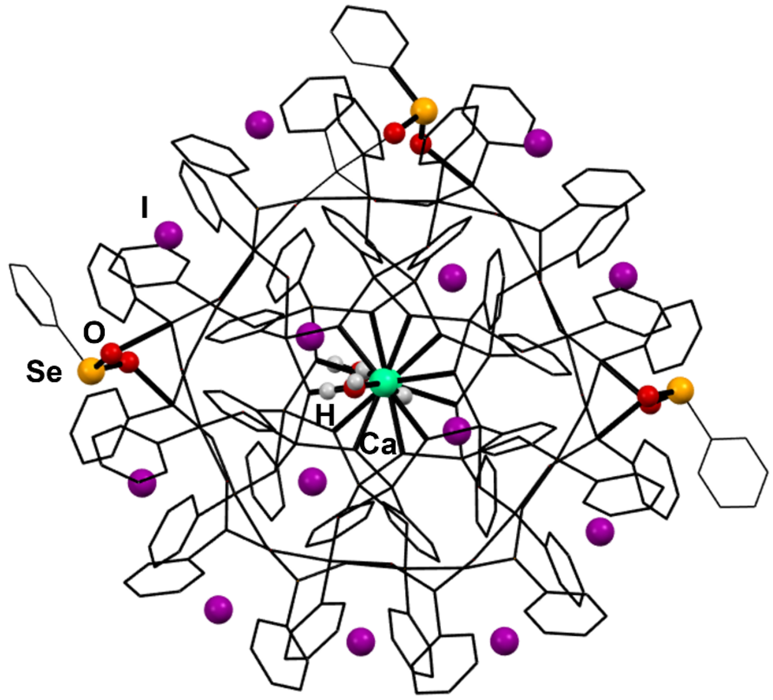

Figure S24. Top-view of the gas-phase optimized structure of $[\{(PhTe)_{18}O_{24}Ca(H_2O)_2)\}_2I_{13}(PhSeO_2)_3]$.

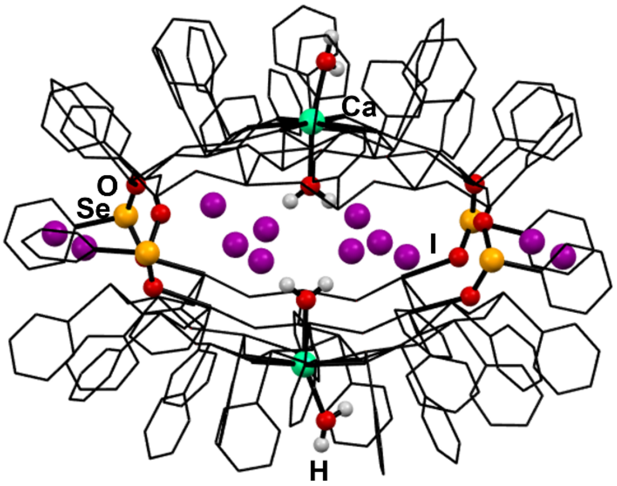

Figure S25. Side-view of the gas-phase optimized structure of [{$(PhTe)_{18}O_{24}Ca$-$(H_2O)_2$)}$_2I_{12}(PhSeO_2)_4$].

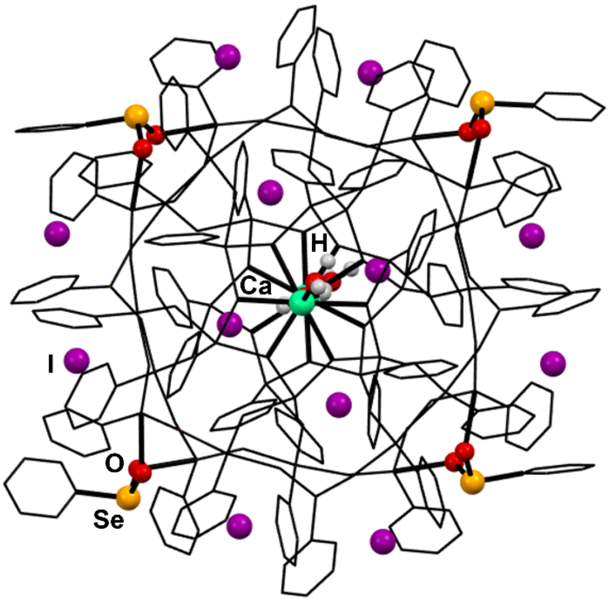

Figure S26. Top-view of the gas-phase optimized structure of [{$(PhTe)_{18}O_{24}Ca$-$(H_2O)_2$)}$_2I_{12}(PhSeO_2)_4$].

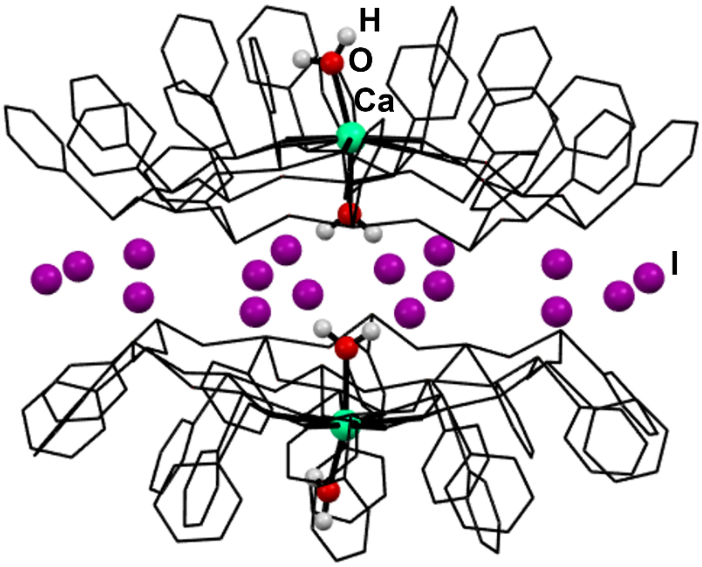

Figure S27. Side-view of the gas-phase optimized structure of $[\{(PhTe)_{18}O_{24}Ca(H_2O)_2\}_2I_{16}]$.[8]

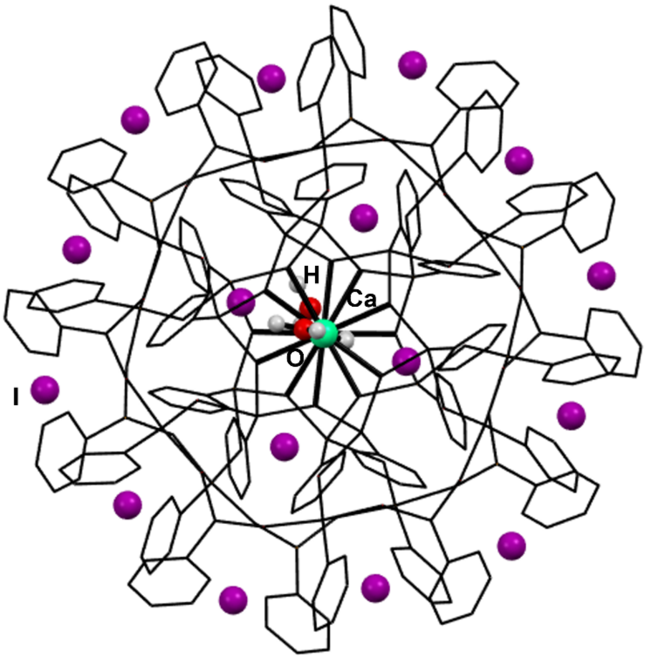

Figure S28. Top-view of the gas-phase optimized structure of $[\{(PhTe)_{18}O_{24}Ca(H_2O)_2\}_2I_{16}]$.[8]

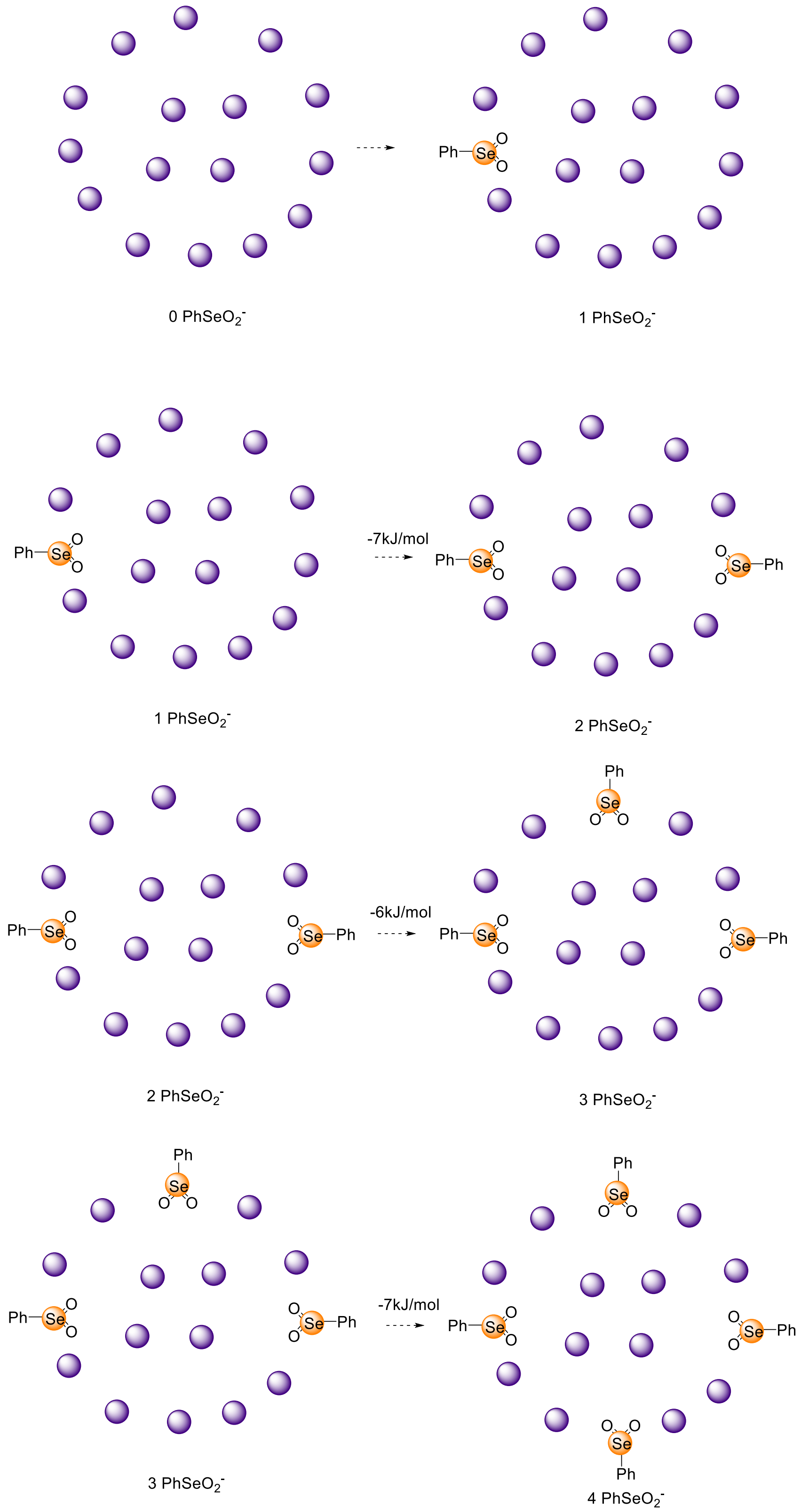

Figure S29. Schematic representation of the considered relative energies of consecutive I→$PhSeO_2$ exchanges.

Table S2. Relative energies of consecutive I→$PhSeO_2$ exchanges.

# of $PhSeO_2^-$	0[8]	1	2	3	4
E [a.u.]	-12756.79	-13135.36	-13513.95	-13892.53	-14271.11
**Definitions of differences according to line 1**		**1-0**	**2-1**	**3-2**	**4-3**
ΔE(I→$PhSeO_2$) [a.u.]		-378.58	-378.58	-378.58	-378.59
**Definitions of differences of differences according to line 1**		**(1-0)-(2-1)**	**(2-1)-(3-2)**	**(3-2)-(4-3)**	
$\Delta\Delta E(I_x \rightarrow (PhSeO_2)_x; I_{x+1} \rightarrow (PhSeO_2)_{x+1})$ [a.u.]		0.0027	0.0025	0.0028	
$\Delta\Delta E(I_x \rightarrow (PhSeO_2)_x; I_{x+1} \rightarrow (PhSeO_2)_{x+1})$ [kJ/mol]		7.1	6.6	7.3	

Table S3. Natural bond orbital (NBO) analysis in [{$(PhTe)_{18}O_{24}Ca(H_2O)_2$)}$_2I_{15}(PhSeO_2)$]: Comparison of the most relevant donor-acceptor orbital interactions and their second-order perturbation stabilization energies $E_{deloc.}$ (kcal/mol), orbital energy differences (au) and Fock matrix element *F*(i,j) between $PhSeO_2^-$ and one exemplar outer shell I^- to the respectively neighboring {$PhTeO_x$}$^+$ units in [{$(PhTe)_{18}O_{24}Ca(H_2O)_2$)}$_2I_{15}(PhSeO_2)$].

	Donor (i)	Acceptor (j)	Interaction	$E_{deloc.}$	$\Delta E_{i,j}$	*F*(i,j)
Se-O···Te	LP(3)O510	LP*(3)Te1	n-n*	51.88	0.32	0.125
Se-O···Te	LP(1)O510	LP*(3)Te1	n-n*	11.94	0.66	0.090
Se-O···Te	LP(3)O511	LP*(2)Te402	n-n*	46.02	0.33	0.121
Se-O···Te	LP(1)O511	LP*(2)Te402	n-n*	12.19	0.66	0.091
I···Te	LP(4)I266	LP*(2)Te256	n-n*	45.60	0.10	0.068
I···Te	LP(3)I266	LP*(2)Te174	n-n*	34.89	0.10	0.059

Table S4. Natural bond orbital (NBO) analysis in [{$(PhTe)_{18}O_{24}Ca(H_2O)_2$)}$_2I_{16}$]: Comparison of the most relevant donor-acceptor orbital interactions and their second-order perturbation stabilization energies $E_{deloc.}$ (kcal/mol), orbital energy differences (au) and Fock matrix element *F*(i,j) between one exemplar outer shell I^- to the two neighboring {$PhTeO_x$}$^+$ units in [{$(PhTe)_{18}O_{24}Ca(H_2O)_2$)}$_2I_{16}$].

	Donor (i)	Acceptor (j)	Interaction	$E_{deloc.}$	$\Delta E_{i,j}$	*F*(i,j)
I···Te	LP(4)I124	LP*(2)Te300	n-n*	8.81	0.09	0.027
I···Te	LP(3)I124	LP*(2)Te300	n-n*	28.77	0.15	0.065
I···Te	LP(4)I124	LP*(2)Te27	n-n*	29.31	0.09	0.050
I···Te	LP(3)I124	LP*(2)Te27	n-n*	17.37	0.15	0.050

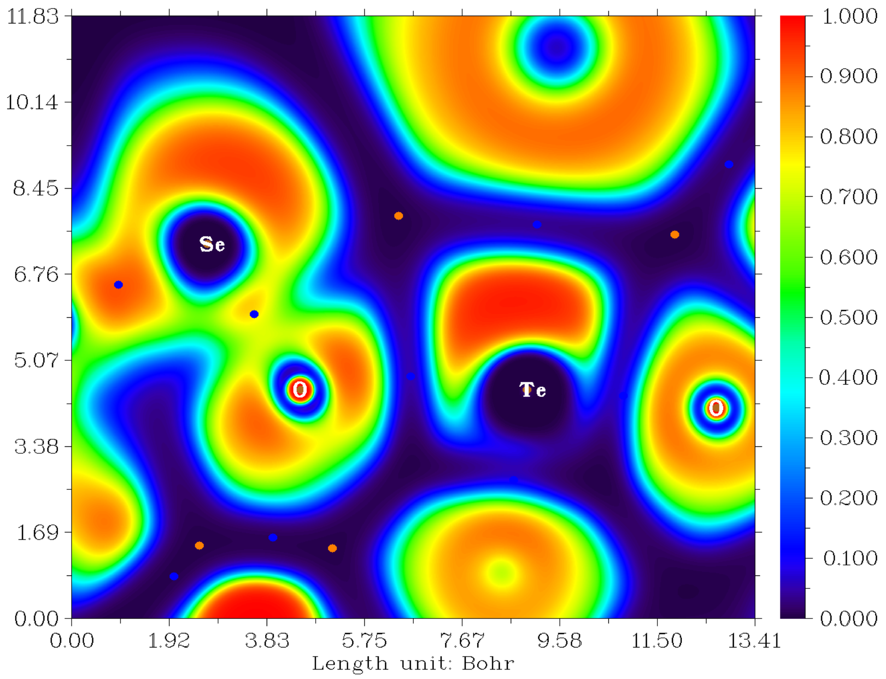

Figure S30. Slice through the electron localization function (ELF) of one exemplar Se-O···Te moiety in [{(PhTe)$_{18}$O$_{24}$Ca(H$_2$O)$_2$)}$_2$I$_{15}$(PhSeO$_2$)]. Critical points from the topological analysis: (3,-3) or "nuclear": brown; (3,-1) or "interaction": blue; (3,1) or "ring": orange.

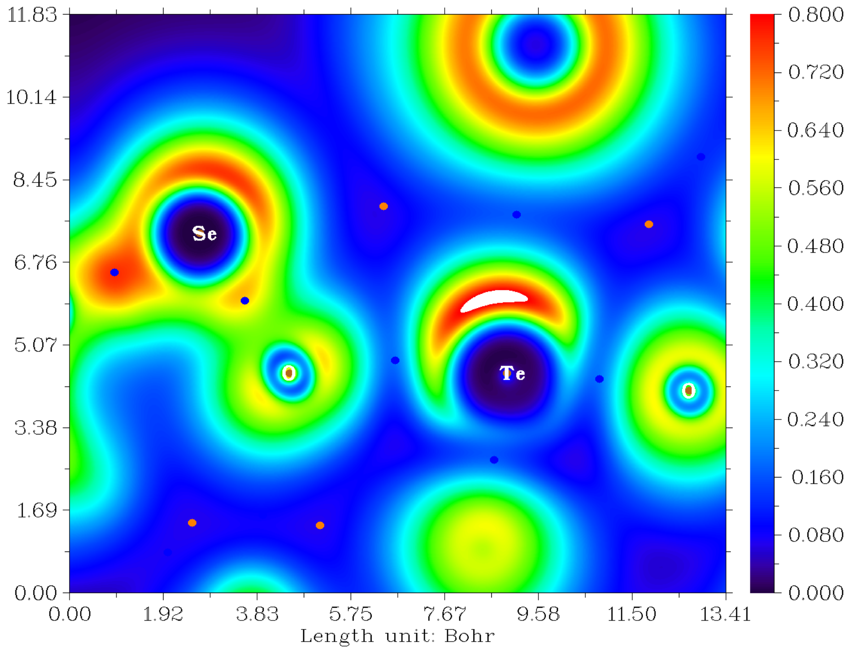

Figure S31. Slice through the localized orbital locator (LOL) of one exemplar Se-O···Te moiety in [{(PhTe)$_{18}$O$_{24}$Ca(H$_2$O)$_2$)}$_2$I$_{15}$(PhSeO$_2$)]. Critical points from the topological analysis: (3,-3) or "nuclear": brown; (3,-1) or "interaction": blue; (3,1) or "ring": orange.

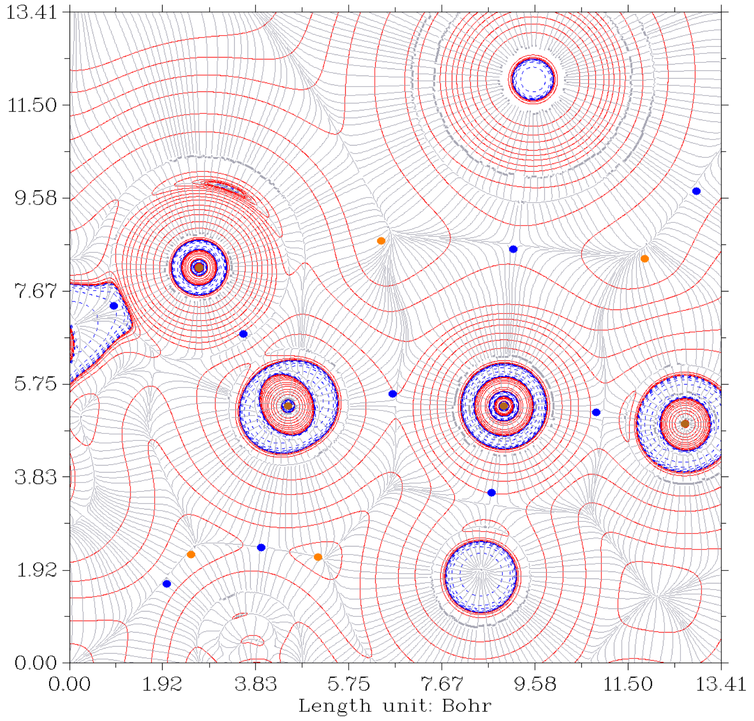

Figure S32. Slice through the Laplacian of the electron density of one exemplar Se-O···Te moiety in [{$(PhTe)_{18}O_{24}Ca(H_2O)_2$)}$_2I_{15}(PhSeO_2)$]. Critical points from the topological analysis: (3,-3) or “nuclear”: brown; (3,-1) or “interaction”: blue; (3,1) or “ring”: orange.

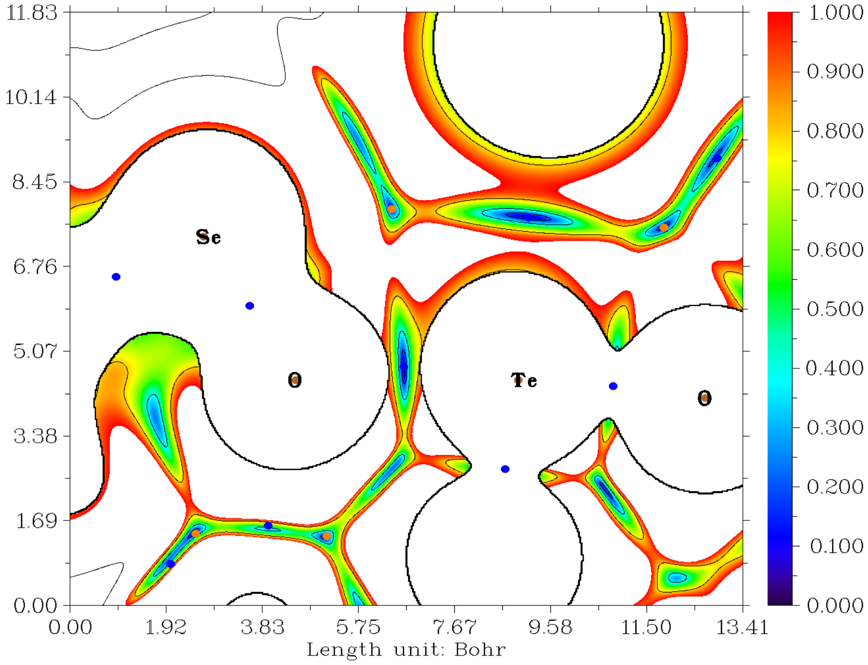

Figure S33. Slice through the reduced density gradient (RDG) of one exemplar Se-O···Te moiety in [{$(PhTe)_{18}O_{24}Ca(H_2O)_2$)}$_2I_{15}(PhSeO_2)$]. Critical points from the topological analysis: (3,-3) or “nuclear”: brown; (3,-1) or “interaction”: blue; (3,1) or “ring”: orange.

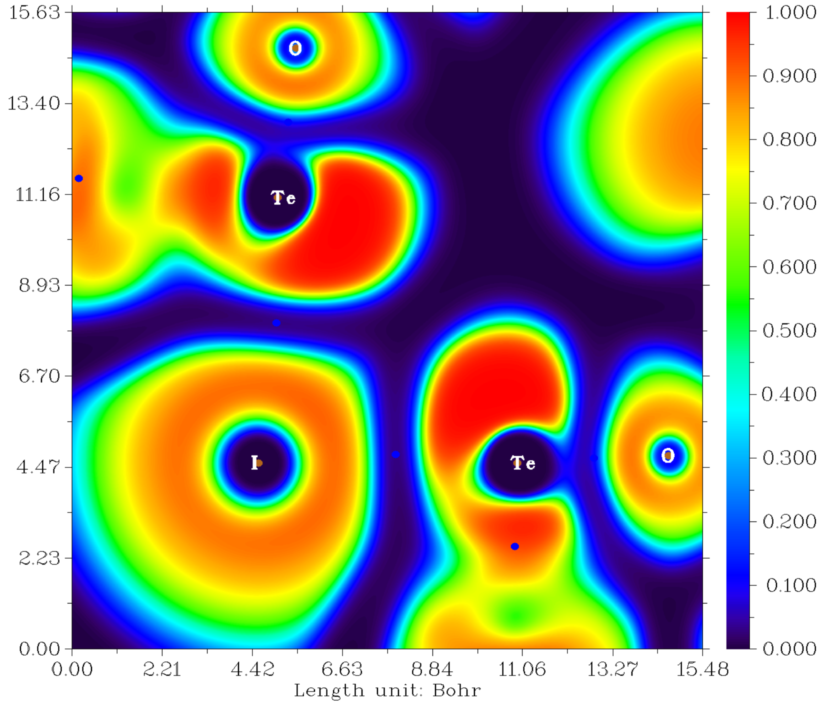

Figure S34. Slice through the electron localization function (ELF) of one exemplar outer Te···I···Te moiety in [{(PhTe)$_{18}$O$_{24}$Ca(H$_2$O)$_2$)}$_2$I$_{15}$(PhSeO$_2$)]. Critical points from the topological analysis: (3,-3) or "nuclear": brown; (3,-1) or "interaction": blue; (3,1) or "ring": orange.

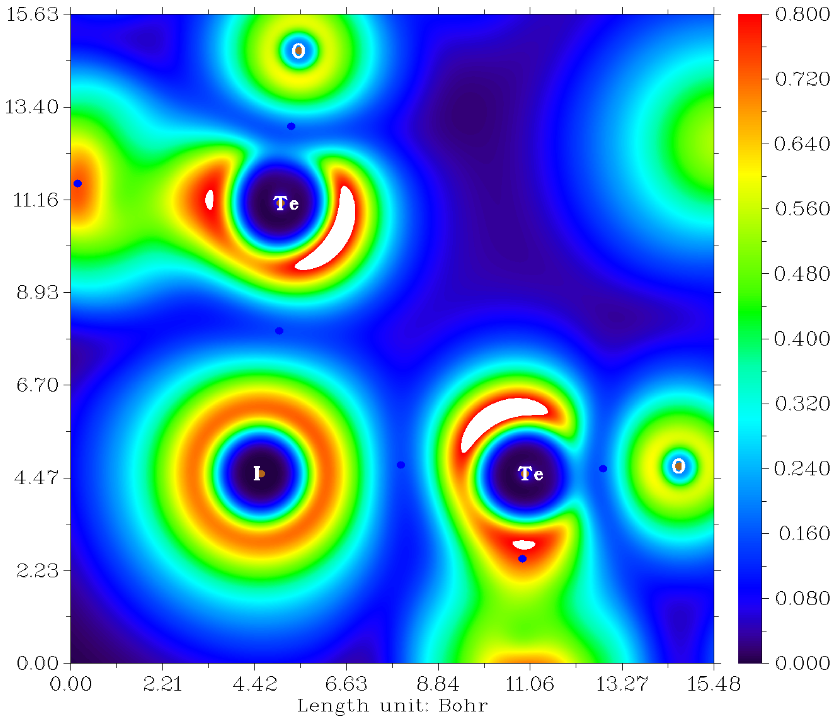

Figure S35. Slice through the localized orbital locator (LOL) of one exemplar outer Te···I···Te moiety in [{(PhTe)$_{18}$O$_{24}$Ca(H$_2$O)$_2$)}$_2$I$_{15}$(PhSeO$_2$)]. Critical points from the topological analysis: (3,-3) or "nuclear": brown; (3,-1) or "interaction": blue; (3,1) or "ring": orange.

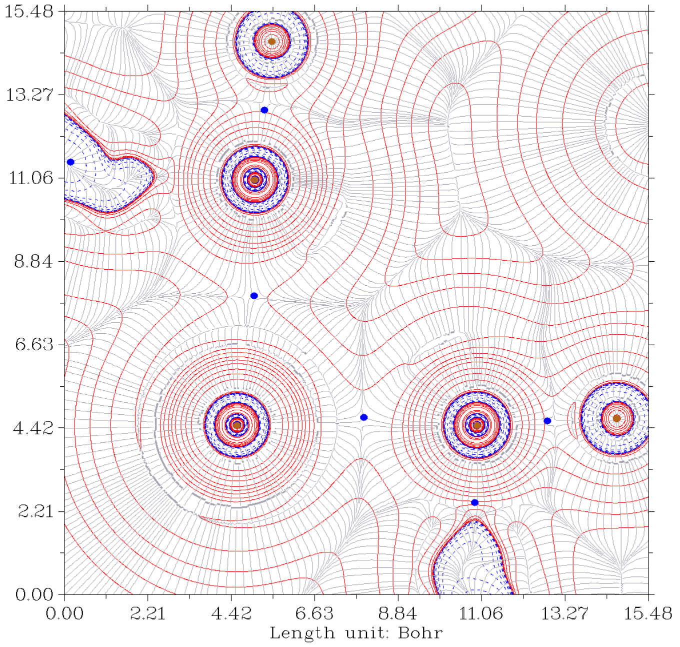

Figure S36. Slice through the Laplacian of the electron density of one exemplar outer Te···I···Te moiety in $[\{(PhTe)_{18}O_{24}Ca(H_2O)_2\}_2I_{15}(PhSeO_2)]$. Critical points from the topological analysis: (3,-3) or "nuclear": brown; (3,-1) or "interaction": blue; (3,1) or "ring": orange.

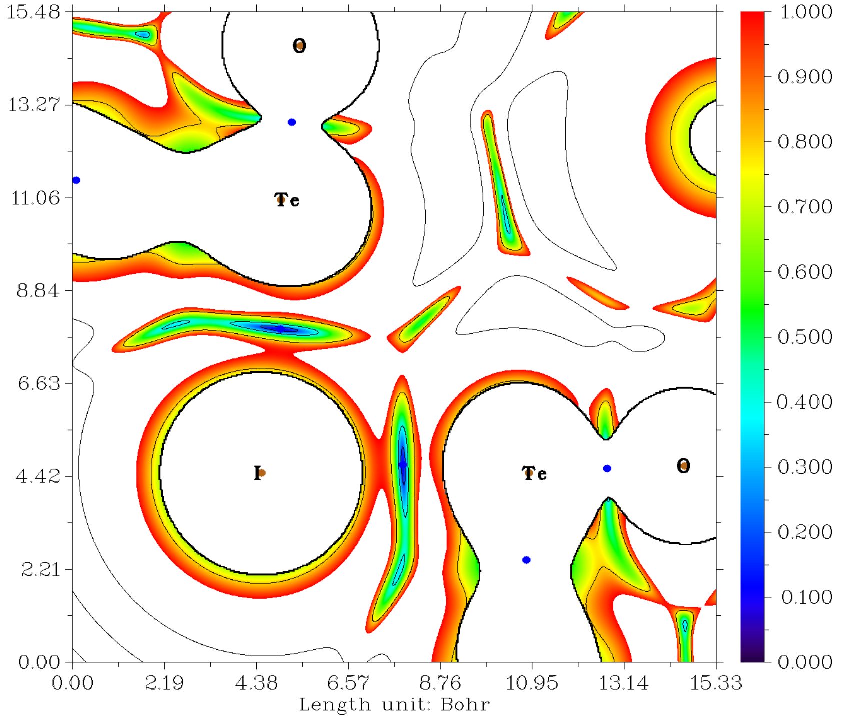

Figure S37. Slice through the reduced density gradient (RDG) of one exemplar outer Te···I···Te moiety in $[\{(PhTe)_{18}O_{24}Ca(H_2O)_2\}_2I_{15}(PhSeO_2)]$. Critical points from the topological analysis: (3,-3) or "nuclear": brown; (3,-1) or "interaction": blue; (3,1) or "ring": orange.

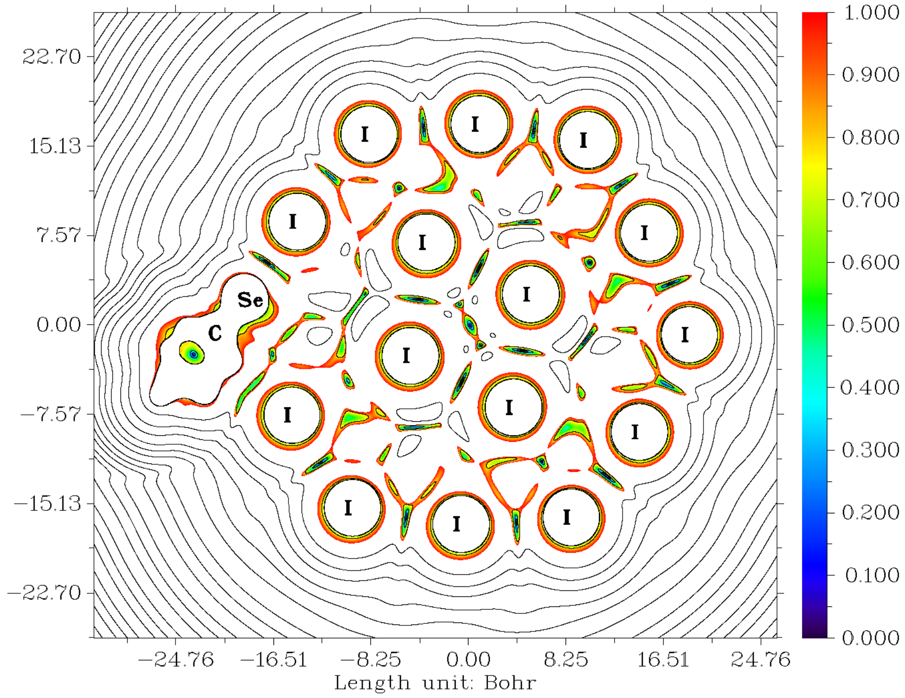

Figure S38. Slice through the reduced density gradient (RDG) in the xy-plane (iodide layer) of $[\{(PhTe)_{18}O_{24}Ca(H_2O)_2\}_2I_{15}(PhSeO_2)]$.

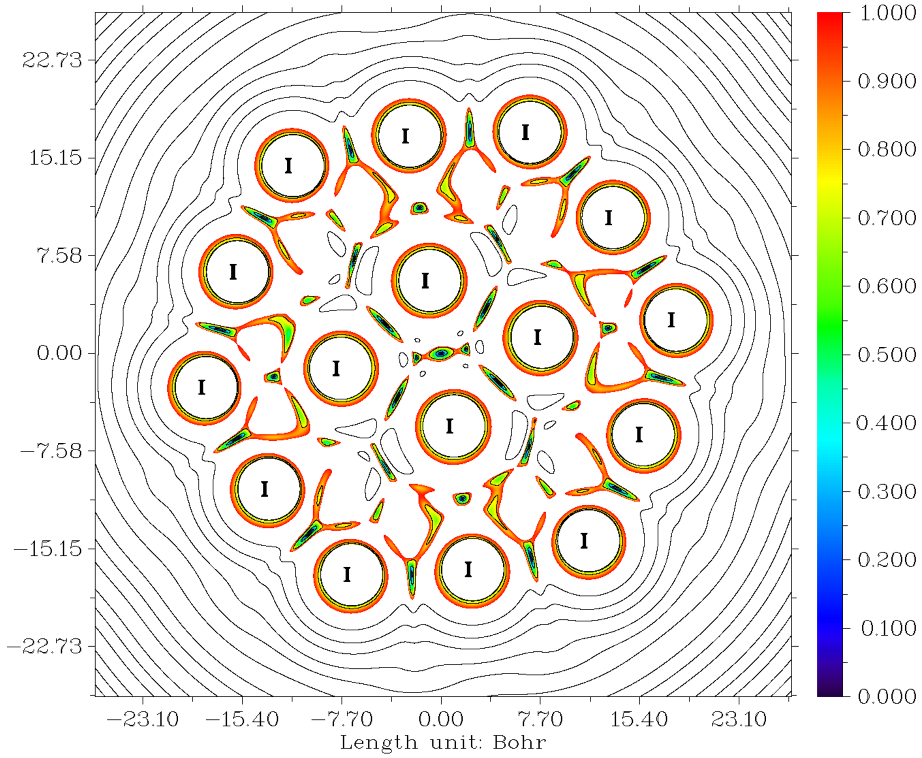

Figure S39. Slice through the reduced density gradient (RDG) in the xy-plane (iodide layer) of $[\{(PhTe)_{18}O_{24}Ca(H_2O)_2\}_2I_{16}]$.[8]

7. References

1. Irgolic, K. C. *Organotellurium Compounds* (Houben-Weyl), *Methods of Organic Chemistry*, Vol. E12b, Georg Thieme, Stuttgart, 1990, pp. 177.
2. McCullough, J. D. The dissociation constants of some mono-substituted benzeneselenic acids. *J. Am. Chem. Soc.* **1949**, *71*, 674 – 676.
3. Sheldrick, G. M. A short history of SHELX. *Acta Crystallogr*. **2008**, *A64*, 112 – 122.
4. Sheldrick, G. M. Crystal structure refinement with SHELXL. *Acta Crystallogr. Section C: Structural Chemistry*, **2015**, *71*, 3-8.
5. Dolomanov, O. V.; Bourhis, L. J.; Gildea, R. J.; Howard, J. A. K.; Puschmann, H. OLEX2: A complete structure solution, refinement and analysis program. *J. Appl. Cryst.* **2009**, *42*, 3339-341.
6. Tyan, Y.-S.; Preuss, D. R.; Vazan, F.; Marino, S. J. Laser recording in tellurium suboxide thin films. *J. App. Phys.* **1986**, *59*, 716-719.
7. M. J. Frisch, G. W. Trucks, H. B. Schlegel, G. E. Scuseria, M. A. Robb, J. R. Cheeseman, G. Scalmani, V. Barone, G. A. Petersson, H. Nakatsuji, X. Li, M. Caricato, A. V. Marenich, J. Bloino, B. G. Janesko, R. Gomperts, B. Mennucci, H. P. Hratchian, J. V. Ortiz, A. F. Izmaylov, J. L. Sonnenberg, D. Williams-Young, F. Ding, F. Lipparini, F. Egidi, J. Goings, B. Peng, A. Petrone, T. Henderson, D. Ranasinghe, V. G. Zakrzewski, J. Gao, N. Rega, G. Zheng, W. Liang, M. Hada, M. Ehara, K. Toyota, R. Fukuda, J. Hasegawa, M. Ishida, T. Nakajima, Y. Honda, O. Kitao, H. Nakai, T. Vreven, K. Throssell, J. A. Montgomery, Jr., J. E. Peralta, F. Ogliaro, M. J. Bearpark, J. J. Heyd, E. N. Brothers, K. N. Kudin, V. N. Staroverov, T. A. Keith, R. Kobayashi, J. Normand, K. Raghavachari, A. P. Rendell, J. C. Burant, S. S. Iyengar, J. Tomasi, M. Cossi, J. M. Millam, M. Klene, C. Adamo, R. Cammi, J. W. Ochterski, R. L. Martin, K. Morokuma, O. Farkas, J. B. Foresman, and D. J. Fox, Gaussian 16, Revision B.01, Gaussian, Inc., Wallingford CT, 2016.
8. Kirsten, L. Rodrigues, J. F.; Hagenbach, A.; Springer, A.; Pineda, N. R.; Piquini, P. C.; Roca Jungfer, M.; Schulz Lang, E.; Abram, U. Large Telluroxane Bowls Connected by a Layer of Iodine Ions. *Angew. Chem. Int. Ed.* **2021**, *60*, 15517-15523.
9. Dennington, R.; Keith, T. A.; Millam, J. M. GaussView, Version 6, Semichem Inc., Shawnee Mission, KS, 2016.
10. Binkley, J. S.; Pople, J. A.; Hehre, W. J. Self-consistent molecular orbital methods. 21. Small split-valence basis sets for first-row elements. *J. Am. Chem. Soc.* **1980**, *102*, 939-947.
11. Dunning, T. H.; Hay, P. J. Gaussian basis sets for molecular calculations in 'Methods of Electronic Structure Theory'. Ed. Schaefer, H. F. *Modern Theoretical Chemistry*, Springer, **1977**.
12. Hay, P. J.; Wadt, W. R. *Ab initio* effective core potentials for molecular calculations. Potentials for K to Au including the outermost core orbitals. *J. Chem. Phys.* **1985**, *82*, 299-310.
13. Wadt, W. R.; Hay, P. J. *Ab initio* effective core potentials for molecular calculations. Potentials for main group elements Na to Bi. *J. Chem. Phys.* **1985**, *82*, 284-298.

14. Check, C. E.; Faust, T. O.; Bailey, J. M.; Wright, B. J.; Gilbert, T. M.; Sunderlin, L. S. Addition of Polarization and Diffuse Functions to the LANL2DZ Basis Set for P-Block Elements. *J. Phys. Chem. A*, **2001**, *105*, 8111-8116.
15. Pritchard, B. P.; Altarawy, D.; Didier, B.; Gibson, T. D.; Windus, T. L. New Basis Set Exchange: An Open, Up-to-Date Resource for the Molecular Sciences Community. *J. Chem. Inf. Model.* **2019**, *59*, 4814-4820.
16. Feller, D. The role of databases in support of computational chemistry calculations. *J. Comput. Chem.* **1996**, *17*, 1571-1586.
17. Schuchardt, K. L.; Didier, B. T.; Elsethagen, T.; Sun, L.; Gurumoorthi, V.; Chase, J.; Li, J.; Windus, T. L. Basis Set Exchange: A Community Database for Computational Sciences. *J. Chem. Inf. Model.* **2007***, 47, 1045-1052.*
18. Lu, T.; Chen, F. Multiwfn: A multifunctional wavefunction analyzer. *J. Comput. Chem.*, **2012**, *33*, 580–592.
19. Johnson, E. R.; Keinan, S.; Mori-Sánchez, P.; Contreras-García, J.; Cohen, A. J.; Yang, W. *J. Am. Chem. Soc.*, **2010**, *132*, 6498-6506.
